# Genome-wide association analysis of *Monilinia fructicola* lesion in a collection of Spanish peach landraces

**DOI:** 10.3389/fpls.2023.1165847

**Published:** 2023-10-23

**Authors:** Pedro J. Martínez-García, Jorge Mas-Gómez, Ángela S. Prudencio, Juan José Barriuso, Celia M. Cantín

**Affiliations:** ^1^ Department of Plant Breeding, Centre of Edaphology and Applied Biology of Segura, Spanish National Research Council (CEBAS-CSIC), Murcia, Spain; ^2^ AgriFood Institute of Aragon (IA2), CITA-Universidad de Zaragoza, Zaragoza, Spain; ^3^ Department of Pomology, Experimental Station of Aula Dei-CSIC, Spanish National Research Council, Zaragoza, Spain

**Keywords:** brown rot, cuticle, disease resistance, hypersensitive response, SNP, Spanish germplasm

## Abstract

Brown rot, caused by the *Monilinia* spp., is the disease that causes the greatest losses in stone fruit worldwide. Currently, *M. fructicola* has become the dominant species in the main peach production area in Spain. The fruit cuticle is the first barrier of protection against external aggressions and may have a key role in the susceptibility to brown rot. However, information on the role of skin fruit on the resistance to brown rot in peach is scarce. Previous genetic analyses in peach have demonstrated that brown rot resistance is a complex and quantitative trait in which different fruit parts and resistance mechanisms are involved. To search for genomic areas involved in the control of the cultivar susceptibility to brown rot and to elucidate the role of fruit skin against this infection, we have studied, for two consecutive seasons (2019 and 2020), the fruit susceptibility to *M. fructicola*, together with fruit cuticle thickness (CT) and density (CD), in a collection of 80 Spanish and 5 foreign peach cultivars from the National Peach Collection at CITA (Zaragoza, Spain). Brown rot incidence, lesion diameter, and severity index were calculated after 5 days of inoculation on non-wounded fruit. The peach collection has also been genotyped using the new peach SNP chip (9 + 9K). Genotypic and phenotypic data have been used to perform a genome-wide association analysis (GWAS). Phenotyping has shown a wide variability on the brown rot susceptibility within the Spanish germplasm as well as on CD and CT. The GWAS results have identified several significant SNPs associated with disease severity index (DSI), CD, and CT, five of which were considered as reliable SNP-trait associations. A wide protein network analysis, using 127 genes within the regions of the reliable SNPs and previously identified candidate genes (169) associated with *Monilinia* spp. resistance, highlighted several genes involved in classical hypersensitive response (HR), genes related to wax layers as ceramidases and lignin precursors catalyzers, and a possible role of autophagy during brown rot infection. This work adds relevant information on the complexity resistance mechanisms to brown rot infection in peach fruits and the genetics behind them.

## 1 Introduction

Brown rot, caused by the necrotrophic fungi *Monilinia* spp. (Ascomycota), is one of the most economically important diseases in stone fruits (Van Leeuwen et al., 2002). In Europe, *M. laxa* and *M. fructicola* have been the most recurrent pathogens since the dislodgement of *M. fructigena* in 2010 (Villarino et al., 2013). Similar to what has also been reported in California peach orchards during the second half of the twentieth century (Hewitt and Leach, 1939; Zehr, 1982; Ogawa et al., 1985), *M. fructicola* is nowadays the predominant brown rot species in the main Spanish peach-growing areas such as the Ebro Valley. In Spain, brown rot infection may cause over 80% fruit loss after harvest (Usall et al., 2015; Villarino et al., 2016). Globally, annual losses due to brown rot have been estimated at 1.7 billion euros (Martini and Mari, 2014), which represents huge economic losses for the growers and an important source of food waste worldwide.

The most common method to control the disease is the application of fungicides at different moments of the fruit cycle (at pre- and postharvest stages). Due to the growing social and political concern about health and environmental sustainability and the more restricted use of fungicides, host tolerance or reduced susceptibility appears as the most efficient and safe strategy to reduce incidence of brown rot in peach growing. Some degree of tolerance to *Monilinia* spp. has been reported in the Brazilian landrace ‘Bolinha’ (Feliciano et al., 1987) and some advanced selections developed by University of California, Davis (Martínez-García et al., 2013a). However, most commercial peach cultivars are susceptible to the infection. On the other hand, as far as we know, there are no previous experiences on evaluating susceptibility to brown rot within the Spanish germplasm.

The Spanish peach germplasm has been genetically characterized in previous works using SSRs and SNPs (Bouhadida et al., 2011; Pérez et al., 2020; Mas-Gómez et al., 2021). The structure of the genetic diversity in this peach germplasm has been described recently, using the high-density Illumina peach 18K SNP v2 array (Mas-Gómez et al., 2021). The population structure was explained by geographic origin and fruit type (Mas-Gómez et al., 2021). Higher values of inbreeding and lower differentiation values were found in northern populations in comparison with southern populations, which may be related to gene flow among proximal northern regions. The genetic diversity observed in this germplasm could contribute to the genetic dissection of important traits and also as important sources of disease resistance.

Brown rot susceptibility in peach has been previously evaluated with different approaches, revealing its polygenic and quantitative inheritance (Pascal et al., 1994; Gradziel et al., 1997; Wagner Júnior et al., 2011; Martínez-García et al., 2013b; Pacheco et al., 2014; Fu et al., 2018; Baró-Montel et al., 2019; Fu et al., 2021; Fu et al., 2022). Initially, QTL studies were carried out using a population based in a peach introgression line from an almond × peach interspecific hybrid source of resistance (Martínez-García et al., 2013b), in an F1 population using as source of resistance the cultivar ‘Contender’ (Pacheco et al., 2014) and in a backcross population using as source of resistance the almond cultivar ‘Texas’ (Baró-Montel et al., 2019). Several QTLs associated with resistance have been previously identified in linkage groups 1, 2, and 4 (Martínez-García et al., 2013b; Pacheco et al., 2014; Baró-Montel et al., 2019). Recently, two genome-wide association studies have been performed using families with ‘Bolinha’, ‘Contender’, and almond as sources of tolerance (Fu et al., 2021; Fu et al., 2022), and SNP-trait associations have been identified in all linkage groups except for linkage group 3. Also, genomic prediction has been explored, although, up to now, only moderate prediction accuracies have been obtained for field disease incidence (Fu et al., 2022).

A relationship between infection susceptibility and cuticle thickness and structure has been suggested for different fruit species such as apple (Konarska, 2012), cranberries (Özgen et al., 2002), and grapes (Marois et al., 1985; Gabler et al., 2003) and immature peaches (Lino et al., 2022). In peach, more tolerant cultivars have been related with thicker cuticles, greater amounts of epicuticular waxes, and higher levels of cell wall components as pectins (Gradziel and Wang, 1993; Crisosto et al., 1997; Gradziel et al., 2002). However, there is a knowledge gap on the fruit cuticle’s physiological role in different processes, such as the defense response against fungal infections. Previous association studies identified candidate genes related to cell wall like pectinesterase (Martínez-García et al., 2013a; Fu et al., 2021), endopolygalacturonases (Baró-Montel et al., 2019), dirigent proteins, peroxidases, and wall-associated receptor kinase genes (Fu et al., 2021), supporting the role of the skin as the primary barrier defense mechanism to brown rot. One important cell wall component is lignin; its biosynthesis and deposition in secondary cell walls performs a physical barrier against initial pathogen infection and is commonly positively correlated with plant disease resistance (Miedes et al., 2014; Wan et al., 2021). Lignin is a product of the phenylpropanoid pathway; such pathway also produces compounds involved in preformed and inducible physical and chemical barriers and in the signaling for defense gene induction (Dixon et al., 2002).

Recognition receptors and signaling are two main processes in the two modes of plant immunity (pathogen-associated molecular patterns (PAMP) and effector-triggered immunity (ETI)) (Tsuda and Katagiri, 2010). Candidate genes for *M. fructicola* recognition and plant immunity response in peach like receptor-like protein kinases and mitogen-activated protein kinases (Martínez-García et al., 2013b), leucine-rich repeat proteins (LRRs), and pathogenesis-related genes transcriptional activator have also been identified (Fu et al., 2021). LRR is the most representative class of plant resistance genes (R) (Dangl and Jones, 2001), participates in specific protein–protein interactions, and is involved in resistance to a diverse range of pathogens (Jones and Jones, 1997). A rapid programmed cell death (PCD) called hypersensitive response (HR) is one of the common responses among those triggered by the activation of R proteins (Wu et al., 2014). The R proteins seem to interact and recruit autophagy-related genes (ATG) to the plasma membrane to initiate autophagy of bacteria entering the host cell. This evidence places autophagy as a crucial element of the innate immune response to intracellular bacteria in humans (Travassos et al., 2010); however, no such clear evidence has been confirmed in peach yet. Autophagy consists in the isolation of cytoplasmic constituents (e.g., proteins) invaginated in a membrane, which later form a double-membrane vesicle called autophagosome that continues toward lysosome or vacuole for degradation (Lai et al., 2011).

Here, we study the susceptibility to brown rot infection within a collection of Spanish peach germplasms for the first time, including new related traits such as the cuticle density and thickness, in order to shed some light on the role of fruit skin in the resistance to infection. The peach collection was phenotyped for fruit response to brown rot using non-wounded protocols and then genotyped with the high-density Illumina peach 18K SNP v2 array (Gasic et al., 2019). Then, we performed a genome-wide association analysis (GWAS) employing single- and multi-locus methods to identify SNP-trait associations. Finally, an enrichment analysis was carried out to find candidate genes associated with the resistance to the infection.

## 2 Materials and methods

### 2.1 Plant material

The genetic diversity in the peach (*P. persica* (L.) Batsch) germplasm from the National Peach Collection at CITA has been described recently, using the high-density Illumina peach 18K SNP v2 array (Mas-Gómez et al., 2021). According to the previous population structure results and the fruit production of each accession, to ensure the posterior phenotyping, a total of 80 Spanish accessions and five foreign ones (‘Andora’, ‘Corona’, ‘Garau’, ‘Paraguayo Francia’, and ‘Pepita’) were selected for this study. Two trees per accession (**Supplementary Table 1**) were studied during two harvest seasons (2019–2020). The CITA peach collection was established in 2010 in Zaragoza, North Eastern Spain (latitude 41 43 42.7 N, longitude 0 48 44.1 W), grafted onto the peach–almond hybrid ‘Garnem’. Fungicide applications were suppressed in this plot after fruit setting.

### 2.2 Phenotyping

Phenotypic evaluations for fruit response to *M. fructicola* infections were performed during two consecutive seasons (2019 and 2020). From each tree, 40 fruits were harvested at commercial maturity and immediately transported to the lab for the assays. The harvest date for each tree was recorded for each season.

#### 2.2.1 Susceptibility to brown rot

Fruit susceptibility to *M. fructicola* (Winter) Honey infection was evaluated by controlled inoculation. The strain of *M. fructicola* used in this study (CPMC3) was provided by the Postharvest Pathology group of IRTA (Lleida, Catalonia, Spain) and was isolated from a latent infection of a peach fruit from a commercial orchard. Out of 40 fruits harvested from each tree, 20 unblemished fruits of similar maturity (0.6–0.8) determined by Index of Absorbance Difference (I_AD_) (Ziosi et al., 2008) were used for inoculations. Non-wounded inoculation was performed by following the protocol of Martínez-García et al. (Martínez-García et al., 2013b). There were 20 non-wounded fruits of each tree (two trees per cultivar) that were inoculated by adding a 10-µL droplet of inoculum with the concentration of 10^5^ conidia per mL of *M. fructicola* isolate CPMC3. Perpendicular brown rot lesion diameters (mm) (BRD) were recorded 5 days after inoculation and incubation of the peaches in the dark humidified containers at 20°C ( ± 1°C). When the lesion diameter was bigger than 70 mm, a maximum BRD of 70 mm was assigned. The disease severity index (DSI) for each cultivar was calculated as the product of the average lesion diameter × proportion of fruit with lesions greater than 10 mm (disease incidence), following protocol described in Fu et al. (2018) with modifications, and averaged for two trees per cultivar at each season. Measurements were done for two consecutive seasons (2019 and 2020).

#### 2.2.2 Cuticle density and thickness

For fruit cuticle assays, three unblemished fruits per tree (and two trees per cultivar) were selected and five epidermal disks (13 mm in diameter) were cut with a cork borer from the equatorial part of each fruit. Maximum amount of flesh was manually removed from each disk, and then cuticles were isolated enzymatically by incubation in pectinase and cellulase buffer, following the protocols of Belge et al. (2014). Isolated cuticles were dried at room temperature for 24 h and then gravimetrically assessed for density calculation. For cuticle observation, 2-mm-thick peach surface pieces were fixed, dehydrated, and embedded in paraffin, following the methodology of Alcaraz et al. (2013). Transverse sections were cut with a microtome and then stained with different dyes (Toluidine Blue + Sudan IV, Sudan IV, and Calcofluor + Auramine O) and then examined by optical microscopy. ImageJ free available software (version 1.47r) was used to calculate cuticle thickness (measured in µm) on microscope images from cuticles stained with Sudan IV (**Supplementary Figure 1**). In each microscope image, an average of the cross section of five points across the cuticle was obtained and averaged, always avoiding areas with trichomes. Three cuticles from three different fruits were measured for each tree (two trees per cultivar). Both density and thickness were measured for two consecutive seasons (2019 and 2020).

#### 2.2.3 Statistical analysis

Statistical analyses were carried out by SPSS Statistics v. 29 (IBM^®^). Frequency histograms were generated with SigmaPlot^®^ v.15 to observe phenotypic data distributions. Normality of the datasets was tested by the Shapiro–Wilk test with a *p*-value threshold of 0.05. Correlation analysis among the datasets was performed using Spearman’s rank correlation coefficients at *p* < 0.05.

### 2.3 Genotyping

DNA samples were genotyped with the new version of the high-density Illumina peach 18K SNP v2 array (Gasic et al., 2019), using an iScan at the “Centre for Research in Agricultural Genomics” (CRAG) in Barcelona (Spain). Genotype calls for each SNP were obtained using the iScan output data in the Genotyping Analysis Module of GenomeStudio™ v2.0.5. (Illumina Inc., San Diego, CA, United States) using the default parameters. SNPs were filtered with the software ASSIsT v1.02 (Di Guardo et al., 2015) establishing a Frequency Rare Allele value of 0.05. SNPs classified as “Monomorphic,” “Failed,” and “NullAllele-Failed” were removed (Di Guardo et al., 2015). Subsequently, the SNPs which overcome the previous step with minor allele frequencies (MAFs) higher than 0.05 were filtered in the Genotyping Analysis Module of GenomeStudio™ v2.0.5 to be used as the high-quality subset of SNPs for further analysis (Vanderzande et al., 2019; Fu et al., 2021).

### 2.4 GWAS

GWAS was performed to find marker-trait associations with the DSI, cuticle density (CD), and cuticle thickness (CT). Single-locus and multi-locus models were used by GAPIT v.3.1. R Package (Lipka et al., 2012) (GLM, MLM, MLMM, BLINK, and FarmCPU) and mrMLM v. 4.0 (Zhang et al., 2020b) (mrMLM, FASTmrMLM, FASTmrEMMA, pLARmEBB, pKWmEB, ISIS, and EM-BLASSO). In all the models, a principal component analysis calculated by the GAPIT package was included as covariate including five principal components. In addition, kinship was calculated and included in analyses performed in the mrMLM package. Three phenotypic datasets corresponding to 2019, 2020, and the average of both years were analyzed. Moreover, those individuals whose DSI was not consistent between years were removed. Significative associations were determined using Bonferroni correction with α = 0.05 in analysis performed in GAPIT and LOD score ≥3 in those performed in mrMLM. Only the associations identified as significant by two models and/or datasets were considered as reliable. The effects caused by reliable SNP associations were predicted using SnpEff v4.3.e (Cingolani et al., 2012). Peach reference genes annotations (v2.0.a1) were used as input, and the effects were categorized by impact (Cingolani et al., 2012). A search of candidate genes in haploblocks containing reliable SNP associations and in those genes subjected to an effect of the reliable SNPs was performed using the reference genome *Prunus persica* v2.1. Haploblocks were obtained using ‘—blocks’ restricted to 1 Mb in PLINK v1.9 (Purcell et al., 2007). Functional annotations of candidate genes were identified using the gene list analysis tool from the PANTHER classification system (Mi et al., 2017). Finally, the candidate genes identified here together with candidate genes detected in previous studies were studied in a protein network analysis using the STRING tool v.11.5 (Szklarczyk et al., 2015). The STRING tool predicts interactions among proteins using multiple sources (genomic context predictions, high-throughput lab experiments, conserved co-expression, automated text mining, and previous knowledge in databases) (Szklarczyk et al., 2015). The sequences were used as input, and clustering of the proteins was implemented using the MCL method and a value of 3 as the inflation parameter.

## 3 Results

### 3.1 Phenotypic data

#### 3.1.1 Brown rot susceptibility

**Figure 1:**
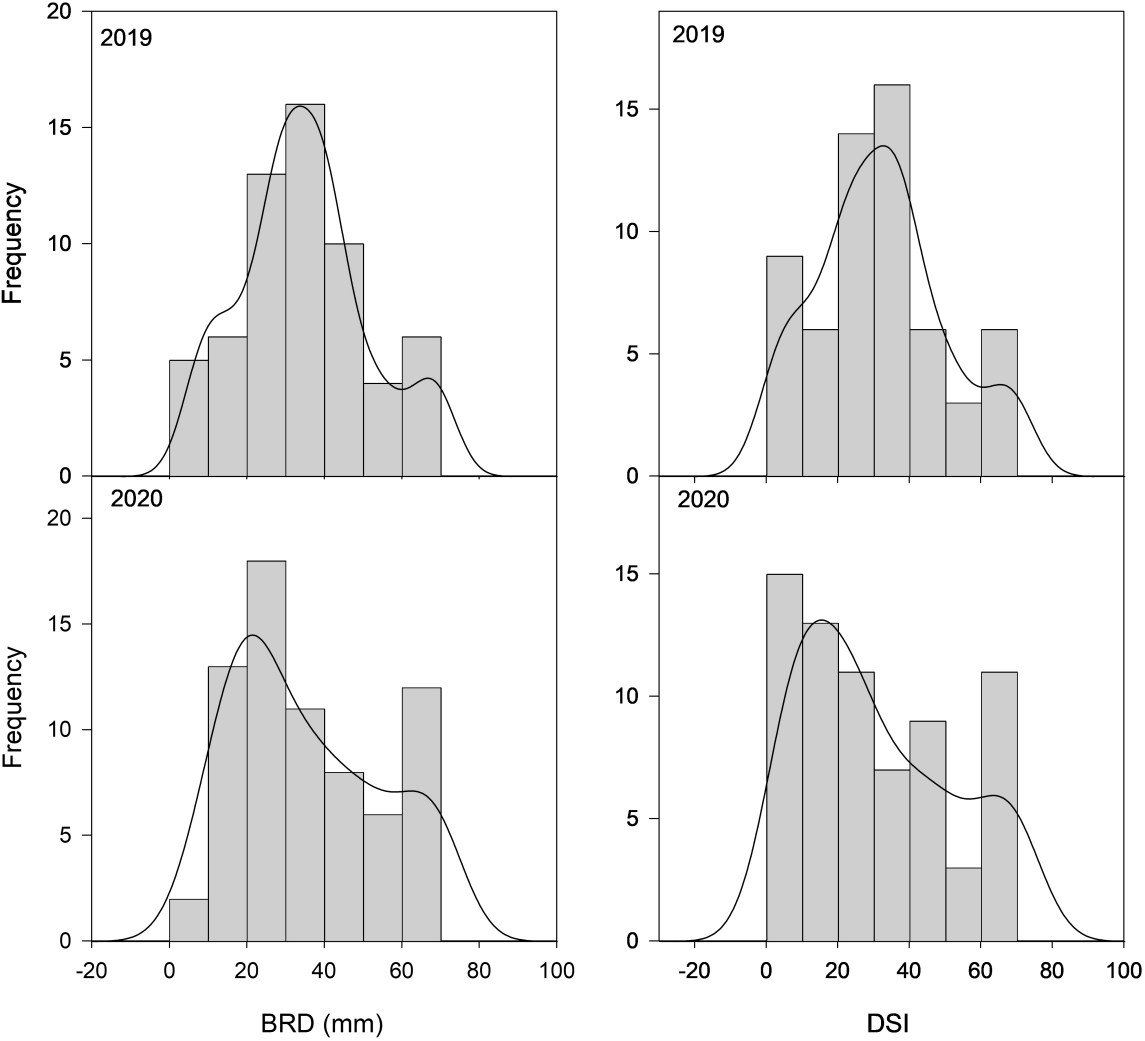
Distribution frequency of 85 peach cultivars from the CITA National peach collection over two consecutive seasons (2019–2020) for brown rot diameter (BRD) and disease severity index (DSI) observed in non-wounded fruit. Frequency (y-axis) is expressed as the number of individuals falling on each phenotypic interval (x-axis).

A total of 80 peach cultivars with Spanish origin and five foreign ones were evaluated for susceptibility to brown rot respecting the integrity of the fruit skin (unwounded fruit) in order to keep the natural resistance to infection associated with fruit skin (**Supplementary Figure 2**). The results of the inoculations showed a significant variability among the peach cultivars studied (**Figure 1**), ranging from 1.35 to 69.37 in 2019 and between 3.65 and 70 in 2020 (**Table 1** and **Supplementary Table 2**). Since a high percentage of cultivars showed low DSI, frequency distribution was skewed toward the left, especially during 2020. A DSI lower than 20 was observed for 15 and 28 cultivars in 2019 and 2020, respectively. On the other extreme, DSI higher than 40 was observed for 15 and 23 cultivars in 2019 and 2020, respectively. The Shapiro–Wilk normality test showed a normal distribution for DSI in 2019 (*p* = 0.106), whereas in 2020 data did not pass the normality test.

**Table 1:** Maximum (max.), minimum (min.), average (ave.), and standard deviation (SD) of phenotypic traits of 85 peach cultivars from the CITA National peach collection in 2019, 2020, and averaged for both consecutive seasons.

Year	Trait	Min.	Max.	Ave	SD
**2019**	BRD	5.91	70.00	34.84	16.46
	DSI	1.35	69.38	29.42	16.86
	CD	12.01	63.40	32.66	11.14
	CT	5.43	8.07	6.67	0.63
**2020**	BRD	4.14	70.00	35.18	19.50
	DSI	3.65	70.00	30.64	21.55
	CD	12.44	48.39	23.67	6.54
	CT	5.99	12.06	8.51	1.21
**Ave. 2019–2020**	BRD	4.14	70.00	35.09	15.38
	DSI	1.59	45.78	19.04	10.57
	CD	12.23	44.43	27.45	7.33
	CT	5.99	12.06	7.97	1.11

BRD, brown rot lesion diameter; DSI, disease severity index; CD, cuticle density; CT, cuticle thickness.

Regarding BRD, all cultivars showed rot diameters between 4 and 70 mm at both seasons, although extreme values (very high and very low) were more abundant in 2020. Only 10 cultivars showed rot diameters higher than 40 mm in 2019, whereas in 2020 there were 18 mm. Similarly to what was observed for the DSI, the Shapiro–Wilk test showed BRD data in 2019 as normally distributed (*p* = 0.097), whereas data in 2020 showed a non-normal distribution.

As observed, although some variation for DSI and BRD was observed between years, most cultivars showed both years a similar trend regarding susceptibility/tolerance to *M. fructicola* infection. Correlation between consecutive years (**Table 2A**) was not significant for BRD, indicating a strong seasonal effect in this trait. However, the correlation was low although significant for DSI (R^2^ = 0.209, *p* < 0.05), suggesting that susceptibility to BR infection could be controlled by a low but significant genetic component.

**Table 2:** Squared Spearman’s rank correlation coefficients in the phenotypic traits of 85 peach cultivars from the CITA National peach collection **(A)**, correlation between years; **(B)**, correlations between traits at each year of study).

a	Trait		Correlation 2019–2020		
	BRD		NS		
	DSI		0.209*		
	CD		0.212*		
	CT		NS		
b			DSI	CD	CT
	**BRD**	2019	0.985*	NS	NS
		2020	0.932**	−0.255**	NS
	**DSI**	2019	1	NS	NS
		2020	1	−0.317**	0.212*
	**CD**	2019		1	NS
		2020		1	−0.165*

BRD, brown rot lesion diameter; DSI, disease severity index; CD, cuticle density; CT, cuticle thickness; NS, not significant; *significant at p < 0.5; **significant at p<0.1.

The red-skin yellow-fleshed non-melting peach ‘Rojo de Tudela’ was the least susceptible cultivar, together with ‘La Escola’ and ‘Gallur’ (both red-skin yellow-fleshed non-melting peaches), whereas the yellow-fleshed non-melting cultivar ‘Borracho de Jarque’ showed the highest susceptibility, closely followed by two types of white-fleshed melting flat peaches (‘Paraguayo Almudí’ and ‘Paraguayo T. Robert’).

#### 3.1.2 Cuticle density and thickness

**Figure 2:**
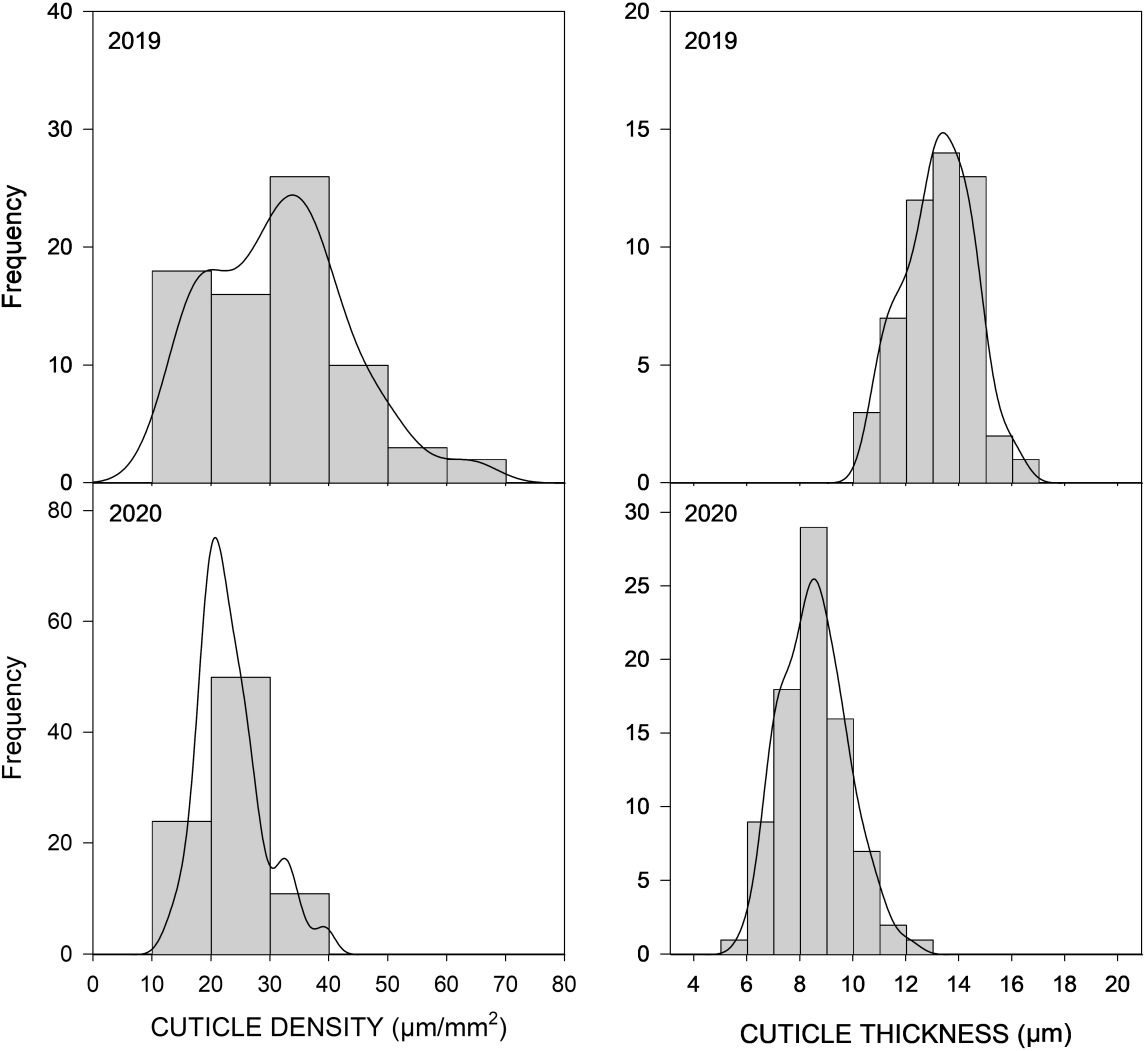
Distribution frequency of 85 peach cultivars from the CITA National peach collection over two consecutive seasons (2019–2020) for cuticle density (CD) and cuticle thickness (CT) observed in non-wounded fruit. Frequency (y-axis) is expressed as the number of individuals falling on each phenotypic interval (x-axis).

Fruit cuticle density (CD) of the 85 peach cultivars ranged between 12.01 and 63.40 µg/mm^2^ in 2019 and between 12.44 and 48.39 µg/mm^2^ in 2020 (**Figure 2**; **Table 1**; **Supplementary Table 1**), which demonstrated the high variability existing on cuticle thickness among different peach cultivars. Cuticle density in the studied cultivars showed a normal distribution, although a slightly higher variability was observed during 2019. In 2019, 15 cultivars exhibited cuticle densities higher than 40 µm/mm^2^, whereas in 2020, no densities higher than 40 µm/mm^2^ were observed. Cuticle density showed a significant although low correlation between years (**Table 2A**, *R* ^2^ = 0.212, *p* > 0.05), suggesting that this trait could have little but significant genetic control.

Cuticle thickness (CT) also showed considerable variability among the peach cultivars studied (**Figure 2**), ranging between 5.43 and 8.07 µm in 2019 and between 5.99 and 12.06 µm in 2020 (**Table 1** and **Supplementary Table 1**). In general, thicker cuticles were observed in 2019 than in 2020. CT was not significantly correlated between years, which could be due to a big seasonal effect on this trait and also to the difficultness for accurate cuticle thickness measurement. The thinnest cuticles were observed in the three clones of the white-fleshed non-melting cultivar ‘Pomar’, with cuticle thickness of approx. 6 µm. On the other hand, the yellow-fleshed non-melting flat-peach ‘Paraguayo Francia’ exhibited the thicker cuticle, with an average of 12.06 µm.

#### 3.1.3 Correlation between traits

Regarding relations between traits (**Table 2B**), as expected, DSI was highly correlated with BRD (0.985 at p < 0.5 and 0.932 at p < 0.1 for 2019 and 2020, respectively), since DSI is calculated with BRD. On the other hand, DSI was significantly correlated with CD and CT in 2020 (−0.317, p < 0.01 and 0.212, p < 0.05, respectively), although correlation was not significant in 2019. On the other hand, BRD was significantly correlated only with CD in 2020 (−0.255, p < 0.01), whereas no correlation was observed with CT for any of the 2 years of study. These results indicate the influence of cuticle on the susceptibility to BR infection, specially its density. Finally, correlation between CD and CT was only slightly significant and negative (−0.165, p < 0.05) in 2020, whereas no significant correlation was observed in 2019. This result may indicate that cuticle density tends to be lower in thicker cuticles. The finding of higher and more significant correlations between traits in 2020 could be due to the higher variability (more extreme values) observed in DSI and BRD in 2020.

### 3.2 Genotyping

A total of 16,038 SNPs was analyzed in GenomeStudio (no data were received from the remaining 1,962 SNPs). ASSIsT determined 1,257 SNPs (7.8%) as failed and 2,798 (17.4%) as monomorphic, and both groups were removed for subsequent analysis (**Supplementary Table 3**). Moreover, 864 SNPs were removed because of a MAF lower than 0.05. Finally, 11,119 SNPs (69.3%) were selected as high-quality SNPs for their use in GWAS. The distribution on chromosomes of the high-quality SNP set range from 707 SNPs on chromosome 5 (6.36%) to 1,951 SNPs on chromosome 2 (17.55%) (**Supplementary Table 4**). Also, the set showed a MAF N50 of 0.294 and a transition/transversion ratio of 3.13.

### 3.3 GWAS

**Figure 3:**
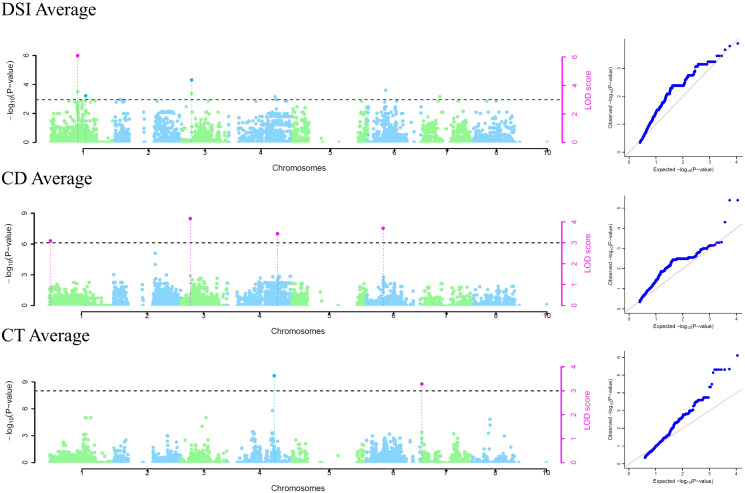
Averaged trait Manhattan plots and QQ plots of significant SNPs of obtained in the mrMLM R package. All the plots per trait/year are in **Supplementary Figures 3**, **4**.

Phenotypic data about DSI of 52 individuals in 2019, 62 individuals in 2020, and 49 in the average dataset were used in GWAS. For studying cuticle density, the 2019 dataset included 76 individuals, 85 individuals in the 2020 dataset, and 76 in the average dataset, and for cuticle thickness the 2019 dataset included 52 individuals, 83 individuals in the 2020 dataset, and 51 in the average dataset. The threshold considering the Bonferroni correction to determine an SNP as significant was 4.5E-06 for the models used in the GAPIT package. The analysis identified a total of 30 significant SNP-trait associations for 19 unique SNPs (**Figure 3**; **Supplementary Figures 3**, **4**; **Supplementary Table 5**) and only by multi-locus models. Eight significant SNP-trait associations were found in the 2019 dataset, 10 in the 2020 dataset, and 12 in the average dataset using mrMLM (**Figure 3**). Nine SNPs significantly associated with DSI were detected in chromosomes 1, 3, 5, and 6. Three of them were identified through two or more models and/or datasets [Peach_AO_0100138 (Chr. 1, 33,041,231 bp); Peach_AO_0309124 (Chr. 3, 240,372 bp); Peach_AO_0648505 (Chr. 6, 15,798,855 bp)], with Peach_AO_0100138 being the SNP identified by the highest number of models (**Table 3**). For cuticle density, seven SNPs significantly associated with the trait were identified in chromosomes 1, 2, 3, 4, and 6, with Peach_AO_0204799 (Chr. 2, 6,467,839 bp) being the unique SNP identified through two or more models (**Table 3**). In the case of cuticle thickness, three SNPs significantly associated with the trait in chromosomes 3, 4, and 7 were obtained and only SNP_IGA_704641 (Chr. 7, 4,178,924 bp) was determined as significant by two models or more (**Table 3**). The allelic effect of the significant SNPs identified through two or more models and/or datasets are represented in **Supplementary Figure 5**. The genotype AA in the SNP Peach_AO_0204799 is associated with a higher cuticle density in 2020 and the genotype GG in SNP_IGA_704641with a higher cuticle thickness in average values. Regarding DSI, the genotype AA in Peach_AO_0100138 in 2019 and average values, the genotype AC in Peach_AO_0309124 in 2020 and average values, and the genotype AA in Peach_AO_0648505 in 2019 showed lower DSI values.

**Table 3:** Reliable SNP-trait associations identified in GWAS.

Trait	SNP	Chr.	Pos. (bp)	Dataset	Model	P value/LOD	r^2^ (%)
**CD**	Peach_AO_0204799	2	6,467,839	2020	FASTmrMLM	3.38	9.03E-08
					ISIS EM-BLASSO	4.19	31.16
**CT**	SNP_IGA_704641	7	4,178,924	Average	FASTmrMLM	3.28	1.87E-05
					ISIS EM-BLASSO	3.28	48.76
**DSI**	Peach_AO_0100138	1	33,041,231	2019	FarmCPU	3.13E-06	–
					FASTmrEMMA	6.43	60.68
					FASTmrMLM	5.96	68.15
					MLMM	1.95E-06	–
				Average	FASTmrEMMA	7.8	69.05
					FASTmrMLM	4.4	21.94
					MLMM	1.36E-08	–
	Peach_AO_0309124	3	240,372	2020	Blink	6.62E-11	–
					MLMM	1.61E-08	–
				Average	FASTmrMLM	4.38	66.14
	Peach_AO_0648505	6	15,798,855	2019	Blink	1.60E-08	–
					ISIS EM-BLASSO	6.60	84.66

The analysis performed in SnpEff showed two moderate effects and eight modifier effects over nine genes (**Supplementary Table 6**). The effects were detected downstream (three effects), intergenic regions (three effects), missense variants (two effects), and upstream (two effects). Three haploblocks containing reliable SNP associations were calculated in the Plink tool (**Table 4**), two of them associated with DSI and one with CD. A total of 127 annotated genes inside of the haploblocks were listed according to the *Prunus persica* v2.1. reference genome (**Supplementary Table 7**). Functional annotations of the candidate genes, together with genes affected identified by SnpEff, were collected using the Panther tool (**Supplementary Table 8**). The amino acid sequences of the 127 annotated genes, together with the amino acid sequences from 169 candidate genes, identified in previous studies to dissect the resistance to brown rot in peach (**Supplementary Table 9**), were imported to STRING to carry out a protein network analysis.

**Table 4:** Haploblocks calculated by Plink which includes reliable SNP-trait associations.

SNP	Trait	Start position haploblock (bp)	Final position haploblock (bp)	Size (bp)
**Peach_AO_0204799**	CD	6,467,778	6,472,902	5,124
**Peach_AO_0100138**	DSI	32,425,512	33,041,231	615,719
**Peach_AO_0648505**	DSI	14,993,445	15,988,023	994,578

**Figure 4:**
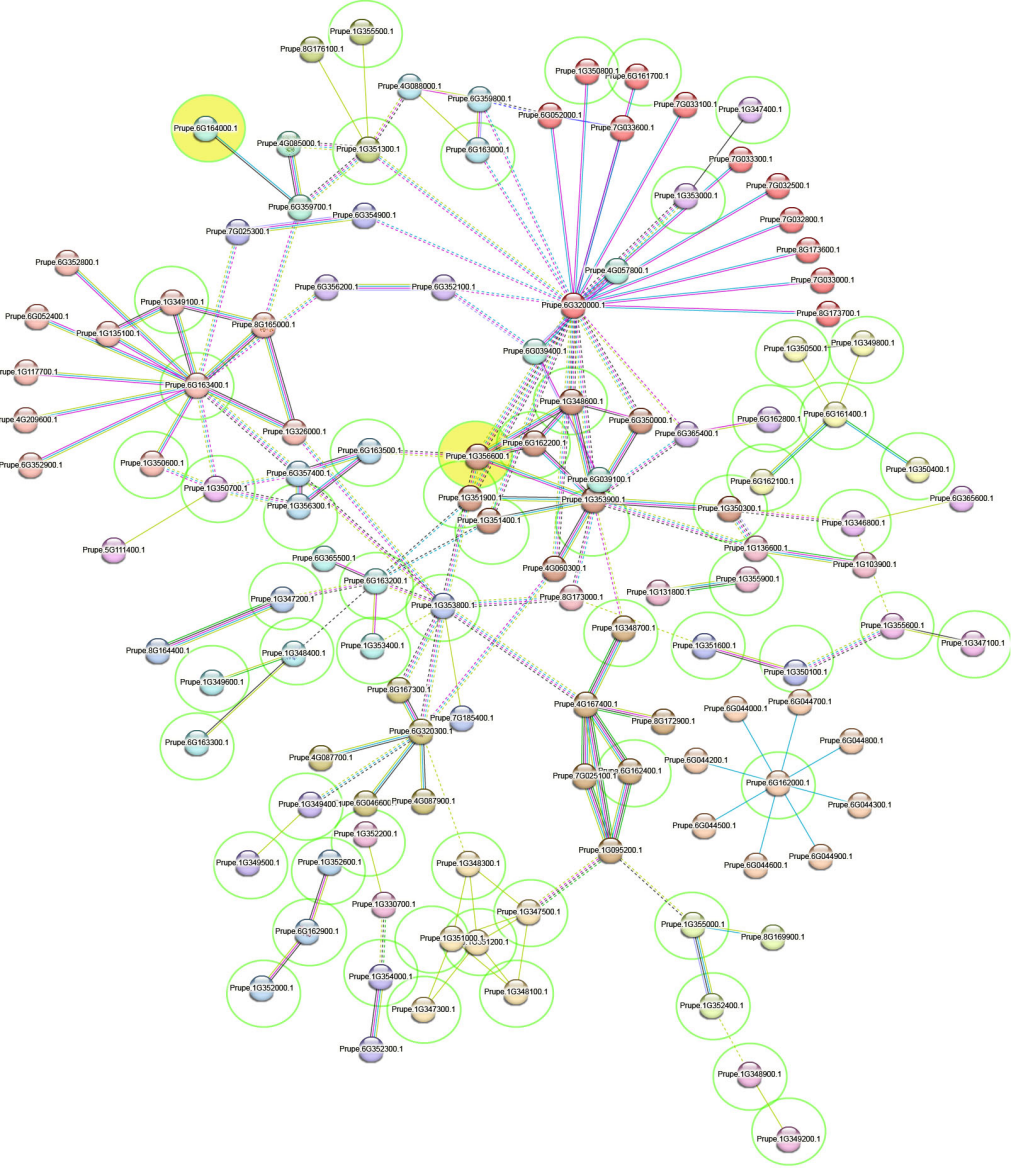
Protein interaction network obtained in STRING (Szklarczyk et al., 2015). Clusters are separated by colors. Those proteins inside a green circle have been identified in the current work, and in yellow-filled ones an SNP effect was identified in SNPeff analysis. The red line indicates the presence of fusion evidence, the green line indicates neighborhood evidence, the blue line indicates cooccurrence evidence, the purple line indicates experimental evidence, the yellow line indicates text mining evidence, the light-blue line indicates database evidence, and the black line indicates coexpression evidence. Inter-cluster edges are represented by a dashed line.

Clustering analysis showed 34 clusters formed of 123 proteins (**Supplementary Table 10**), including 60 protein genes of those identified here (**Figure 4**) and the size of the clusters ranged from 12 (cluster 1) to 1 protein (cluster 34). The functional enrichment in the protein network included 51 functional terms (**Supplementary Table 11**) of Biological Process (Gene Ontology; 8 terms), Molecular Function (Gene Ontology; 9 terms), Cellular Component (Gene Ontology; 1 term), Local network cluster (STRING; 5 terms), KEGG Pathways (1 term), Annotated Keywords (UniProt; 1 term), and Protein Domains (Pfam, 2 terms; InterPro, 14 terms; SMART, 1 term). The functional terms with the highest significance were Secreted (KW-0964), Transmembrane helix (KW-1133), Membrane (KW-0472), Hydrogen peroxide (KW-0376), Peroxidase active site (IPR019794), and Haem peroxidase (IPR002016). Moreover, the complete list of annotations of all the proteins included as input was collected (**Supplementary Table 12**).

## 4 Discussion

This work represents the first work on *M. fructicola* susceptibility on a collection of heirloom Spanish peach cultivars. Several traits such as DSI or cuticle density were evaluated, and for the first time, a trait such as cuticle thickness was evaluated in peach. A comprehensive genetic dissection of these complex traits was performed using the high-density Illumina peach 18K SNP v2 array. Historically, different studies have been published to understand the resistance to this disease in peach, showing few similarities in the genomic regions identified. Clearly, confirming the complex genetic architecture underlying the resistance to this important disease in peach.

*Monilinia* spp. can infect fruit without naturally occurring entry points (García-Benitez et al., 2016) at any developmental stage, although brown rot susceptibility increases dramatically during maturation (Guidarelli et al., 2014). The importance of fruit skin as a first resistance barrier against different pathogens including *Monilinia* spp. has been demonstrated by several studies (Gradziel and Wang, 1993; Pacheco et al., 2014; Oliveira Lino et al., 2016). Also, many previous studies have shown a much higher severity of infection after wounding the fruit (Martínez-García et al., 2013b; Pacheco et al., 2014; Fu et al., 2018; Fu et al., 2022), which demonstrates the defense role of fruit skin against infections. Indeed, ‘Bolinha’ resistance to brown rot has been mostly attributed to its skin (Gradziel and Wang, 1993). That is why, in this work, the susceptibility to brown rot was evaluated by maintaining the integrity of the fruit skin (unwounded fruit) in order to study the natural resistance to infection associated with fruit skin. The values obtained for the disease severity index (DSI) were in the same range as in previous results obtained with non-wounded disease assays and similar protocols (same period of incubation and inoculum concentration) (Pacheco et al., 2014; Baró-Montel et al., 2019). However, we found DSI values higher than those reported by Fu et al. (2021), which might be due to the different virulence of the *M. fructicola* strain used in both works and small differences (such as temperature or humidity) in the infection protocol.

On the other hand, significant although low seasonal correlation was observed in this work for DSI, whereas brown rot diameter (BRD) showed non-significant correlation between years. These low correlation values between years for BR infection traits have been already previously reported by other authors (Pascal et al., 1994; Pacheco et al., 2014; Fu et al., 2021) when using the unwounded protocol. In agreement with our results, Fu et al. (2021) also found that DSI showed a slightly better correlation between years than BRD. These results indicate the high effect of environmental conditions in this infection and also its complex nature. Also, small differences on the BR susceptibility assay (such as the position of the inoculum droplet on the fruit, or small differences on the temperature and HR of the environment on the chamber) could have led to the higher DSI values obtained in the second year of study (2020).

Regarding the peach cuticle density, although previous works are scarce, Belge et al. (2014) found a cuticle density for ‘October Sun’ and ‘Jesca’ cultivars of 16 and 17 µg/mm^2^, respectively, similar to the range obtained here. However, in our study, some cultivars showed twofold higher densities such as ‘Maruja Tejar’, ‘Montaced’, ‘Montamar’, and ‘Sudanell’. CD showed a low but significant correlation between years, whereas CT was not significantly correlated between years, which may indicate that CT is more environmentally dependent than CD. As far as we know, no previous data of cuticle thickness analysis in a group of peach cultivars have been previously reported.

Significant correlations between CD and CT with BRD and DSI found in this study, indicate the importance of cuticle, specially the cuticle thickness, on the fruit defense to brown rot infection. The relationship between susceptibility to different fungus and cuticle thickness and structure has been already reported by previous studies in apple (Konarska, 2012), cranberries (Özgen et al., 2002), and grapes (Marois et al., 1985; Gabler et al., 2003). In agreement with our findings, other authors have related higher tolerance in some peach cultivars to thicker cuticles, greater amounts of epicuticular waxes, and higher levels of cell wall components as pectins (Gradziel and Wang, 1993; Crisosto et al., 1997; Gradziel et al., 2002). In a recent study, Lino et al. (2022) demonstrated that, at the early stage of fruit growth (stage I), surface conductance (which estimates the integrity and deposition of the cuticle on the fruit surface) is related to susceptibility to *M. laxa* infection. As far as we know, there are no other studies in peach were the direct influence of fruit cuticle properties to the brown rot susceptibility is studied. Our results introduce one more variable to have into account when selecting for resistant peach cultivars to brown rot.

The high-density Illumina peach 18K SNP v2 array (Gasic et al., 2019) used here for genotyping has been useful not only in previous association studies in world-wide peach germplasm but also in Spanish germplasm. The SNP distribution on chromosomes was similar to the distribution in the whole chip and the ratio transitions/transversions was higher (3.13) than those obtained in previous studies in peach that are around 1.5 (Martínez-García et al., 2013a; Fresnedo-Ramírez et al., 2013) mainly because of a reduction in transversions that were removed in the design of the array (Verde et al., 2012). Regarding GWAS methodology, although single-locus methods are also appropriate to detect significant marker-trait associations for complex traits, we found significant-trait associations only by multi-locus methods. This result could be related with the size of the effect of each loci identified. In this sense, single-locus approaches seem to be more appropriate in cases involving loci with large effects (Wang et al., 2016; Zhang et al., 2019; Fu et al., 2021).

**Figure 5:**
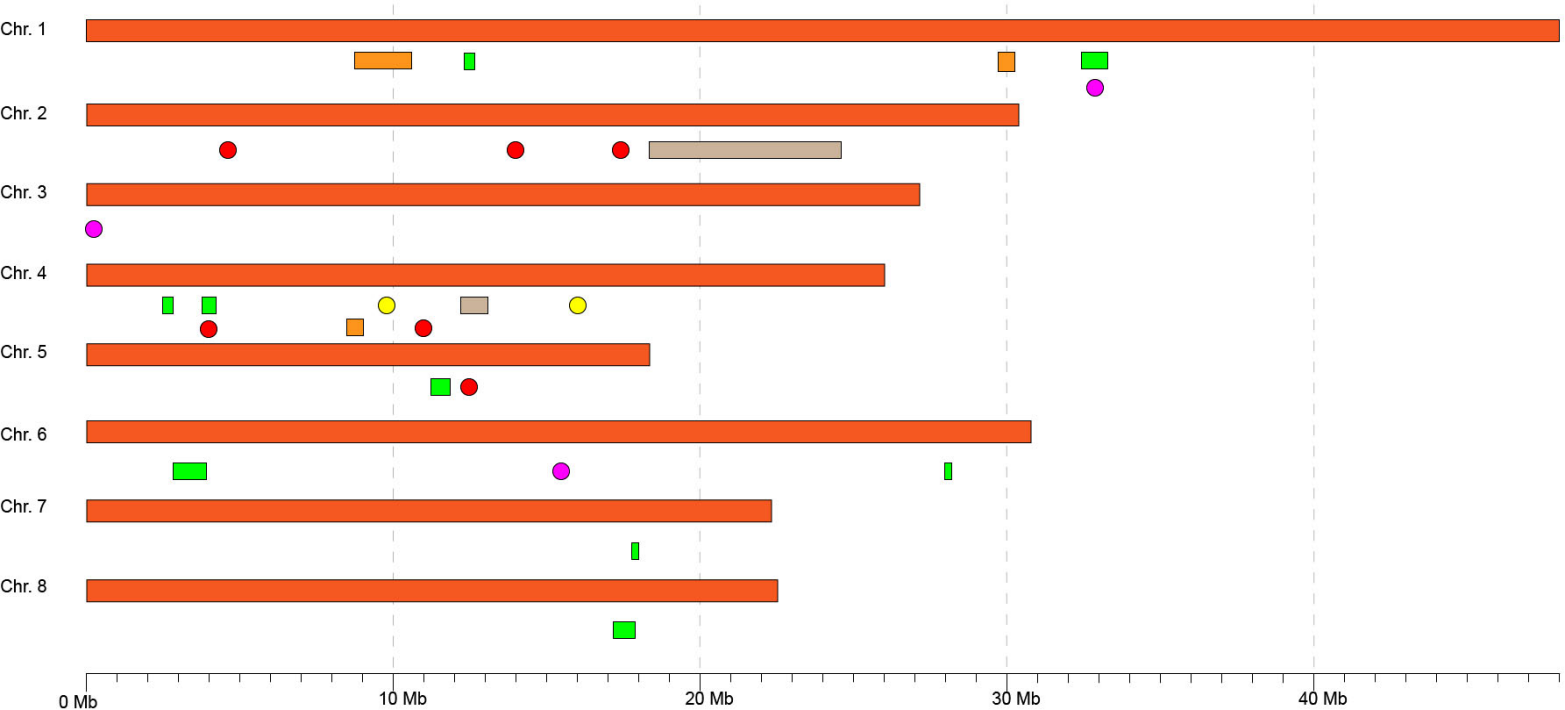
Schematic representation of genomic regions associated with peach non-wounded brown resistance in the current and previous studies. Physical positions of QTLs/significant markers have been obtained from publications or if it was not possible mapping the primer sequence of the marker in the reference genome. The colors represent the study, being orange (Martínez-García et al., 2013b), gray (Pacheco et al., 2014), yellow (Baró-Montel et al., 2019), green (Fu et al., 2021), red (Fu et al., 2022), and pink (the current study).

As has been commented above, although an extensive number of association studies for brown rot resistance have been completed in peach (Martínez-García et al., 2013b; Pacheco et al., 2014; Baró-Montel et al., 2019; Fu et al., 2021; Fu et al., 2022), few genomic regions associated with this resistance are common among studies (**Figure 5**). Moreover, the most robust resistance regions have been identified through unwounded inoculations, which clearly indicates the importance of fruit skin as a first barrier against the fungi. A consensus region is placed around 30–33 Mb in chromosome 1 (**Figure 5**), where a significant SNP has been identified in the current work (Peach_AO_0100138, identified by more methods and/or datasets). This SNP is located near the position of Peach_AO_0100564 (Chr. 1, 33,210,000 bp), a previous SNP associated with brown rot resistance in a peach collection based in a Brazilian source of resistance (Fu et al., 2021) and to a QTL (QTL 1.2) using a population based in a peach introgression line from an almond × peach interspecific hybrid source of resistance (Martínez-García et al., 2013b). Another common region in chromosome 4 (from 9 Mb to 16 Mb) has been identified in brown rot peach resistance studies (Martínez-García et al., 2013b; Pacheco et al., 2014; Baró-Montel et al., 2019; Fu et al., 2022); however, in the current work, no significant SNP has been detected in the region (**Figure 5**). This region is inside of a reported QTL hotspot region in Prunus (7–20 Mbp, Chr.4), where QTLs for quality, agronomic, and disease resistance traits have been reported (Gasic, 2020). These facts reinforce the importance of this region and its future dissection for peach breeding.

Regarding the other two SNPs identified here [Peach_AO_0309124 (Chr. 3, 240,372) and Peach_AO_0648505 (Chr. 6, 15,798,855)], no significant association has been identified in these regions in previous studies, which may suggest the existence of specific favorable alleles in the genetic background of the Spanish germplasm. In addition, two new regions in Chromosome 2 (6,467,839 bp) and Chromosome 7 (4,178,924 bp) were associated with cuticle density and thickness traits for the first time in peach. Indeed, this is the first time that these two traits are included in a GWAS for brown rot susceptibility. The different genetic backgrounds, sources of resistance, and environmental effects together with the polygenic nature may be the causes of the low consistency among studies, even in different datasets in the same study (Martínez-García et al., 2013b; Fu et al., 2021; Fu et al., 2022).

Several candidate genes related to plant disease resistance family genes inside of haploblock containing the reliable SNPs, and protein interactions among them and candidate genes detected in previous studies were identified. The *Prupe.6G161700* gene is annotated as a probable leucine-rich repeat (LRR) receptor-like protein kinase by STRING. LRRs have been previously found in enrichment analysis in peach brown rot resistance association studies (Martínez-García et al., 2013b; Fu et al., 2021). Indeed, the largest cluster detected in the protein interaction analysis grouped mainly LRR-related proteins and 23 proteins of the network showed LRR domain superfamily functional annotations including *Prupe.6G163000*, *Prupe.1G349400*, and *Prupe.6G161700* from the current work. Functional annotations were obtained with the Panther tool revealed as *Prupe_1G351100*, *Prupe_1G350900*, *Prupe_6G163700*, and *Prupe_1G353700* ubiquitin-related proteins, and a conserved protein modifier that linked to target proteins (ubiquitination) leads to their proteasomal degradation, commonly by the 26S proteasome (Marino et al., 2012; Sadanandom et al., 2012). The ubiquitin-26S proteasome system participates from pathogen recognition to downstream signaling (Marino et al., 2012) and also has an important role in the regulation of NB-LRR R protein-mediated plant defense (Cheng and Li, 2012). Although the mentioned genes did not show protein interactions in STRING analysis, a candidate gene detected previously (*Prupe.6G052000*) showed annotations of LRR and ubiquitin-like domain protein and protein interactions with LRR-related proteins of other candidate genes (**Supplementary Table 12**).

Lastly, SNPeff results showed that the reliable SNP associated with DSI in linkage group 6 (Peach_AO_0648505) caused a modifier effect in the intergenic region of the gene *Prupe.6G164000*. According to the functional annotation, this candidate gene belongs to the gene family HOM04D002130, is orthologous to *AT3G18770* and is described as autophagy-related protein 13 (ATG13). Peach has only two genes from this gene family, in chromosome 2 (*Prupe.2G322400*) and chromosome 6 (*Prupe.6G164000).* In this sense, the functional annotation, after the evaluation of the genome architecture of several isolates of *Monilinia* spp., indicated that *Monilinia* spp. infection activated multiple pathways involved in carbohydrate catabolism or autophagy for effective colonization (Akhoon et al., 2023). The autophagy-related proteins (ATGs) ATG1 and ATG13 form a protein kinase complex that regulates autophagosome formation. In *Arabidopsis*, *ATG13* (*ATG13a* and *ATG13b*) seems to be subject to ubiquitylation and proteasomal degradation in different conditions (nutrient starvation or during recovery) (Qi et al., 2020). In 2005, Liu et al. (2005) linked the activation of autophagy to infection in plants and nicely demonstrated that autophagy contributes to resistance. In *Arabidopsis*, autophagy is induced by infection of the necrotrophic fungal pathogen *Botrytis cinerea* and *Arabidopsis* autophagy mutants exhibited enhanced susceptibility to the necrotrophic pathogens *B. cinerea* and *Alternaria brassicicola* (Lai et al., 2011; Lenz et al., 2011). In another study in humans, to understand the pathogenesis of Crohn’s disease, an R protein homolog, the cytosolic NOD1 and NOD2 receptors interacted with ATG16L1 to initiate autophagy of bacteria entering the host cell (Travassos et al., 2010). The role of autophagy in plant resistance seems to be clear (Liu et al., 2005), but at the same time autophagy components and mechanisms might be specifically targeted by pathogen effector proteins to either suppress defense responses or promote pathogenicity, for example, of necrotrophic pathogens (Hofius et al., 2011). In our study, candidate genes associated with autophagy, proteasome, and ubiquitination have been detected. However, the role of autophagy in the skin defense mechanisms against brown rot should be more deeply studied to understand the complexity of the resistance to this important disease in peach in the future.

Candidate genes related to primary barriers family genes were also identified, which could participate in the mechanism of brown rot resistance to avoid *Monilinia* spp. infection. Membrane (KW-0472) was one of the highest significant terms in the enrichment of protein network analysis including 85 proteins, which may reflect the overall importance of the barriers in peach brown rot resistance. Firstly, a moderate effect caused by the reliable SNP Peach_AO_0100138 produces a change of amino acid (missense variant) over the gene *Prupe.1G356500* (Chr.1, 33,039,669 bp). Functional annotations of *Prupe.1G356500* described it as a ceramidase. Ceramidases are enzymes which hydrolyze ceramide and have been related with the regularity of wax layer and susceptibility to *Pseudomonas syringae* in *Arabidopsis* (Wu et al., 2015) and plant resistance to the herbivore *Spodoptera exigua* (Huang et al., 2022). In this line, greater amounts of epicuticular waxes were identified in the epidermis of resistant peaches to brown rot disease (Gradziel et al., 2002). Also, the protective effect of ethylene against *Penicillium digitatum* in ‘Navelate’ oranges has been attributed to the synthesis of new waxes, which imposes a physical barrier to infection (Cajuste et al., 2010). Likewise, n-alkanes and triterpenoids from the cuticular waxes in Asian pears have demonstrated an inhibiting effect on the growth of *Alternaria alternata* (Yin et al., 2011).

On the other hand, the unique KEGG pathway identified in the functional enrichment of the protein network was phenylpropanoid biosynthesis (pper00940), including nine proteins. Phenylpropanoid metabolism was associated with apple fruit resistance to gray mold disease caused by *Botrytis cinerea* because of the contribution of phenolic compounds as chlorogenic and ferulic acid by direct or indirect ways (Ma et al., 2018). High levels of chlorogenic and caffeic acids were observed also in peach breeding lines resistant to brown rot (Bostock et al., 1999; Gradziel et al., 2002). In addition, a significant increase in the transcripts of phenylpropanoid biosynthesis pathway genes was observed studying *Monilinia fructicola* infection in *Arabidopsis* (El-kereamy et al., 2011). Also, the inhibition of postharvest brown rot in peach was achieved inducing the activation of the phenylpropanoid metabolism with the application of nitric oxide (Li et al., 2017). Recently, Kumar Patel et al. (2020) have proved that postharvest application of phenylalanine, a precursor of the phenylpropanoid pathway, to mango and avocado fruit reduced anthracnose and stem-end rot caused by *Colletotrichum gloeosporioides* and *Lasiodiplodia theobromae*, respectively, by inducing a natural defense response.

Eight of the nine proteins involved in the phenylpropanoid pathway were detected previously and were annotated as peroxidase proteins; the remaining one has not been previously observed in earlier studies in peach and was functionally annotated as a cinnamyl-alcohol dehydrogenase (CAD), according to the STRING tool. CAD is a NADP(H)-specific oxidoreductase, which catalyzes the last step in the biosynthesis of monolignols, the monomeric units of the lignin (Singh et al., 2010; Wang et al., 2013). Peroxidase is the enzyme following CAD in the phenylpropanoid pathway and catalyzes the polymerization of phenylpropanoid precursors of lignin and participates in the last step of lignin formation (Singh et al., 2010). Lignin works as a glue, filling the empty spaces in the cellulose–hemicellulose–pectin network (Kärkönen and Koutaniemi, 2010) and thickening the middle lamella and the secondary cell walls of plants, which contributes to cell wall integrity during enzymatic cell wall degradation, pathogen infection, and exposure to abiotic stress (Tian et al., 2016; Liu et al., 2018; Vaahtera et al., 2019). The biosynthesis of monolignols (lignin monomers) is regulated by genes whose transcript abundance significantly improves plant defense against fungi (reviewed by Ninkuu et al. (2023)). Specifically, in fruits, an important role of the peroxidase activity has been observed on lignification in the resistance of apple fruit to *Penicillium expansum* (Valentines et al., 2005). Also, a rise of the lignin content was observed during inoculation of *Botrytis cinerea* in apples, suggesting its role in the inhibition of the gray mold infection (Zhang et al., 2020a). Recently, Yan and Khan (2021) have demonstrated that *Trichoderma harzianum*, a plant fungicide to control *Botrytis*, *Fusarium*, and *Penicillium* spp., induces immunity in tomato through increased expression of CAD, PAL, C4H, and CCOMT for lignin, flavonoid, and phenol accumulation.

For the first time, brown rot resistance in peach and the role of skin cuticle on it has been studied in a large Spanish germplasm and its genetic control has been explored. Brown rot resistance is a complex trait, with a considerable environmental effect and tedious phenotyping process. We have demonstrated for the first time that peach skin cuticle is the first barrier against *M. fructicola*, and therefore selecting cultivars with thicker and denser cuticles could be one more factor to take into account in the combination of factors needed to select for more resistant cultivars to brown rot infection. We carried out a GWAS using different single- and multi-locus models identifying three reliable SNPs associated with DSI in linkage groups 1, 3, and 6. The low consistency in the DSI-associated genomic regions identified in previous studies in different genetic backgrounds has been discussed here, identifying the current consensus regions among studies. Moreover, SNPs associated with cuticle thickness and density traits, the first barrier against brown rot infection, have been identified in linkages group 7 and 2, respectively, being the first report identifying markers associated with these defense traits. The combination of more than a decade of results to understand the genetic control of brown rot resistance and the new results obtained here highlighted a complex network of genes and proteins, reflecting the expected multilayered innate immune system to prevent brown rot invasion and proliferation. From the primary barriers’ perspective, candidate genes related to wax layers, as ceramidases, and to cell wall components, as lignin precursors catalyzers (peroxidases and cinnamyl-alcohol dehydrogenase) have been identified and could be considered as candidate genes for marker-assisted breeding (MAS). In addition, our results suggest that autophagy could contribute in some way (positively or negatively) in the resistance to brown rot, participating in the defense response or in the pathogenicity associated with this disease in peach. This work highlights the importance of preserving traditional germplasm collections, where favorable alleles can be retrieved to improve brown rot resistance in peach, and provides new information for breeding programs in order to advance toward the selection of less susceptible cultivars to brown rot.

## Data availability statement

The datasets presented in this study can be found in online repositories. The names of the repository/repositories and accession number(s) can be found in the article/**Supplementary Material**.

## Author contributions

Conceptualization, PM-G and CC; methodology, PM-G, JB, and CC; investigation, JM-G, AP, CC, and PM-G; data curation, PM-G, CC, and JM-G; writing—original draft preparation, JM-G, AP, JB, CC, and PM-G; writing—review and editing, JM-G, PM-G, and CC. All authors contributed to the article and approved the submitted version.
