# Supplementary material for: Genome-wide association analysis of Monilinia fructicola lesion in a collection of Spanish peach landraces
Source: Front Plant Sci. 2023 Oct 23;14:1165847. doi: 10.3389/fpls.2023.1165847 (PMC10626550; doi:10.3389/fpls.2023.1165847)
Supplement: Supplementary file 1 [file DataSheet_1.docx]

Supplementary Material

Genome-wide association analysis of *Monilinia fructicola* lesion in a collection of Spanish peach landraces

**Pedro J. Martínez-García^1†^, Jorge Mas-Gómez^1†^, Ángela S. Prudencio^1^, Juan Barriuso^2^ and Celia M. Cantín^2*^**

^1^Department of Plant Breeding, CEBAS-CSIC, 30100 Murcia, Spain

^2^Department of Pomology, Experimental Station of Aula Dei-CSIC, Spanish National Research Council, Apdo. 13034, 50080 Zaragoza, Spain

*** Correspondence:**Celia M. Cantín
[cmcantin@eead.csic.es](mailto:cmcantin@eead.csic.es)


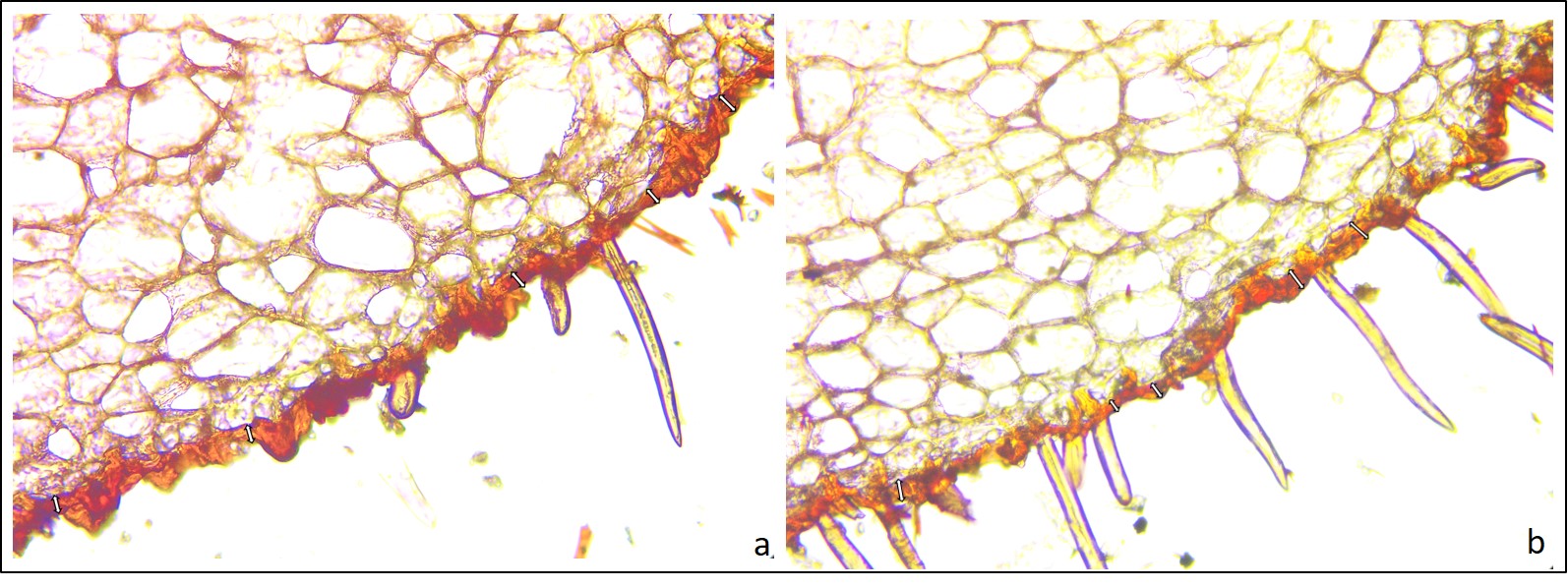


**Supplementary Figure 1**. Images of cross section embedded cuticle tissue of two peach cultivars (a, ‘Sudanell’; b, ‘Fulla’) stained with Sudan IV 0.5% (w/v) and observed by optical microscopy (40x) used for the measurement of cuticle thickness. Five different measurements (white arrows) were taken across the cuticle cross section at each microscope image.


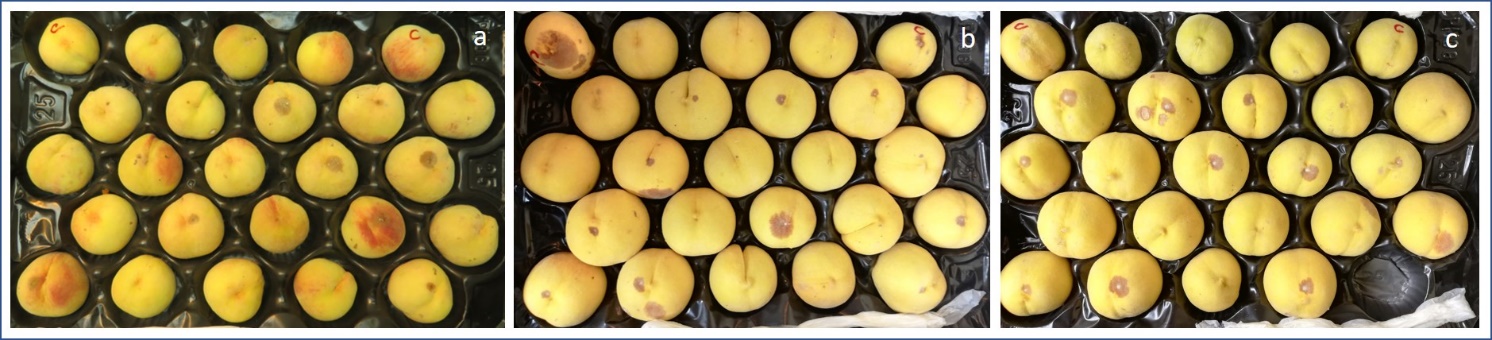


**Supplementary Figure 2.** Brown rot lesions observed in three peach cultivars from the Spanish germplasm collection at CITA (a, ‘Tambarria’; b, ‘Miraflores Serapio’; c, ‘Miraflores’), 5 days after controlled inoculation with *M. fructicola* (strain ‘CPMC3’) and incubation in dark humidified containers at 20˚C (±1˚C).

| 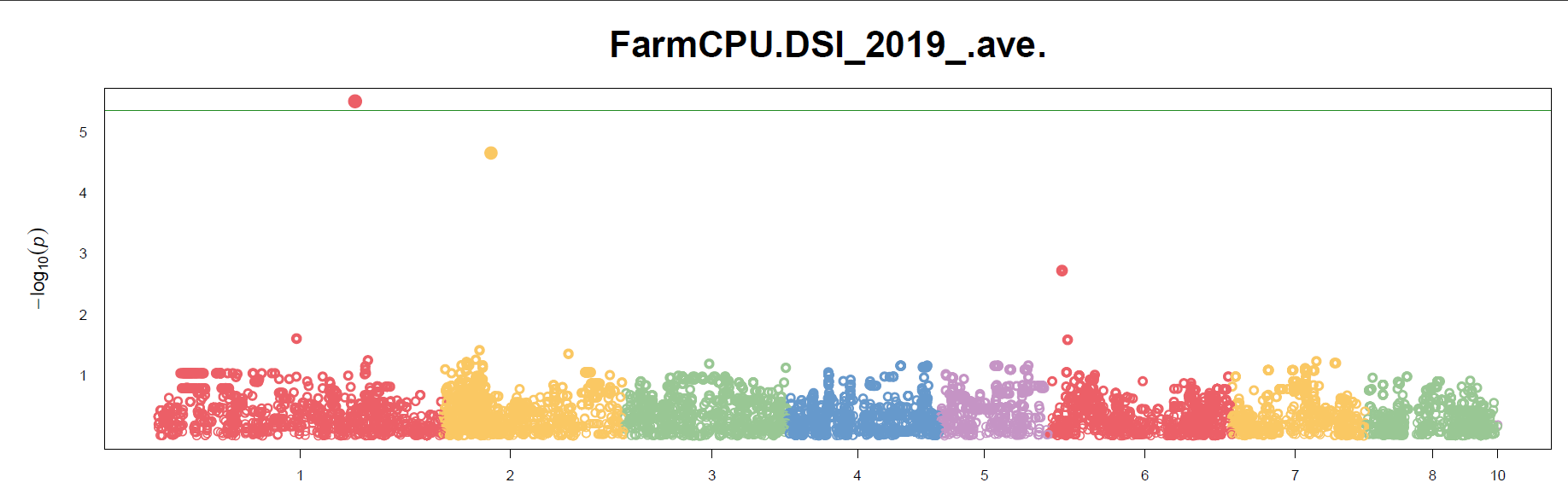 | 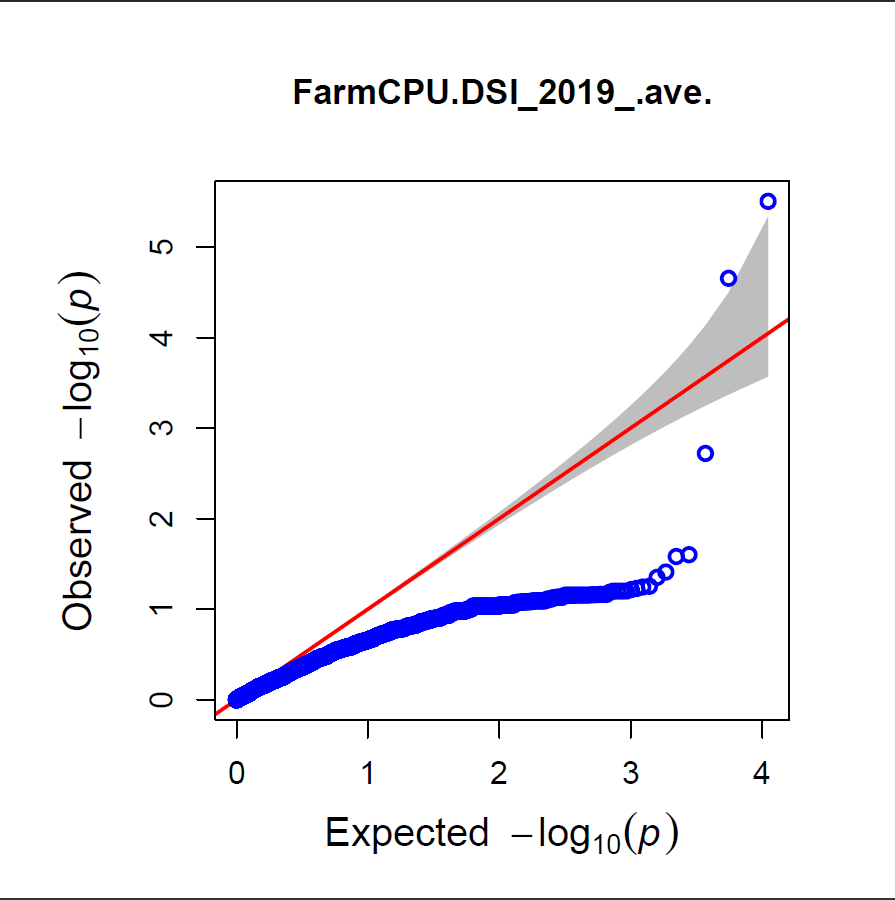 |
| --- | --- |
| 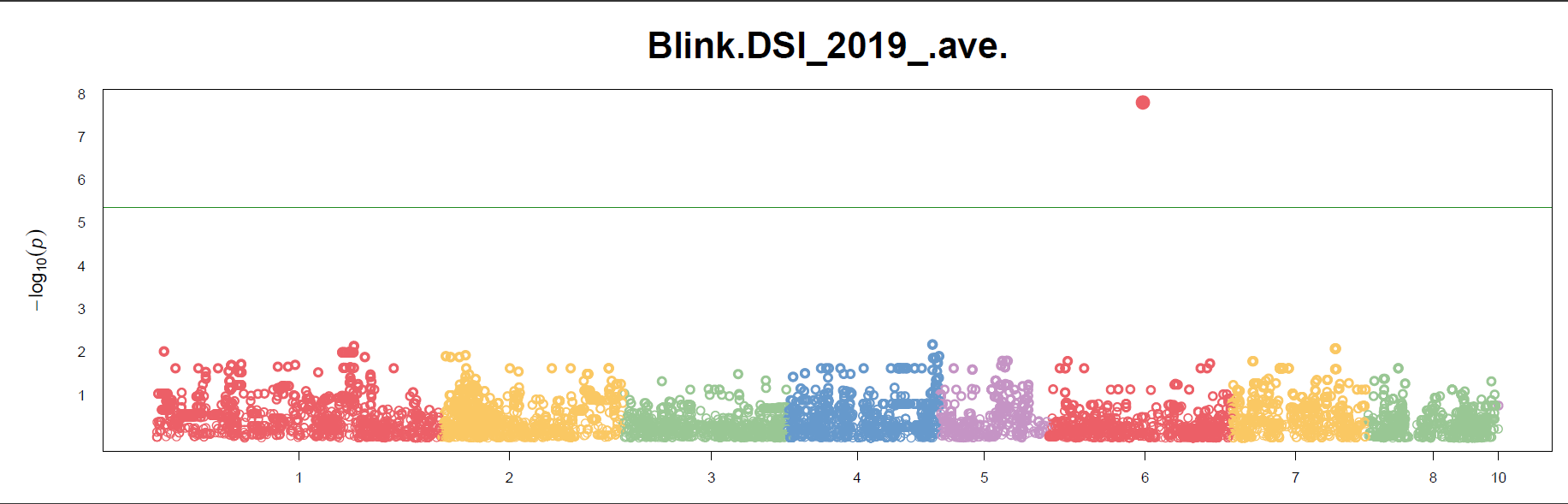 | 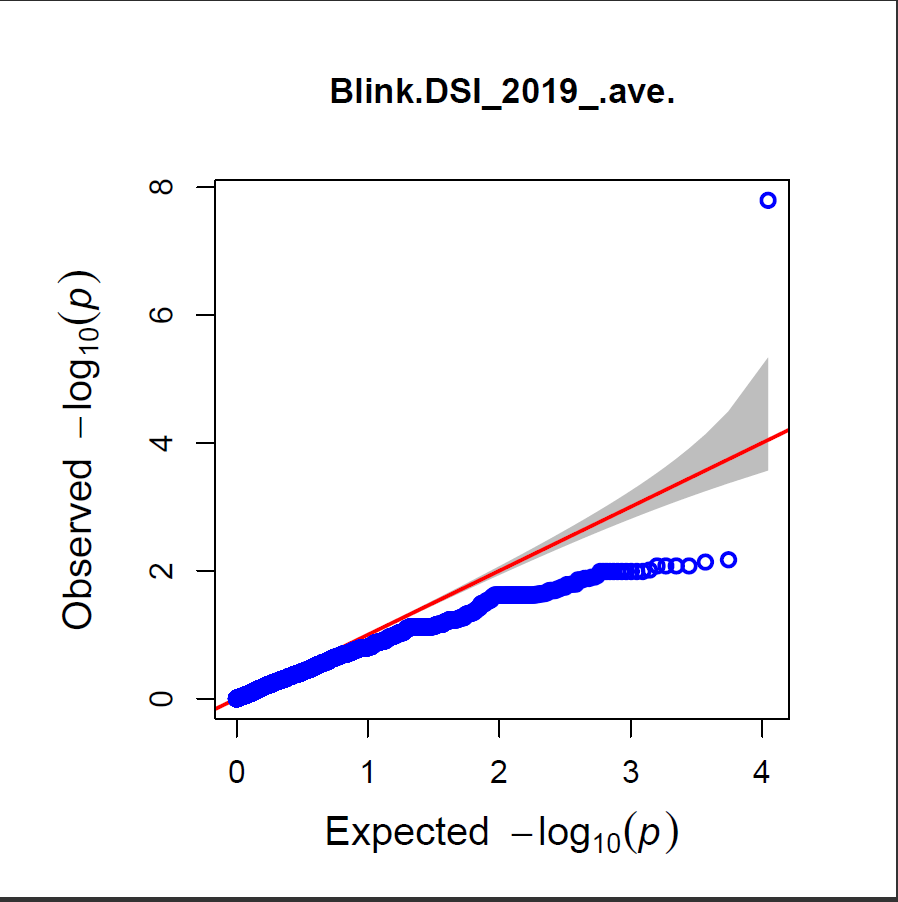 |
| 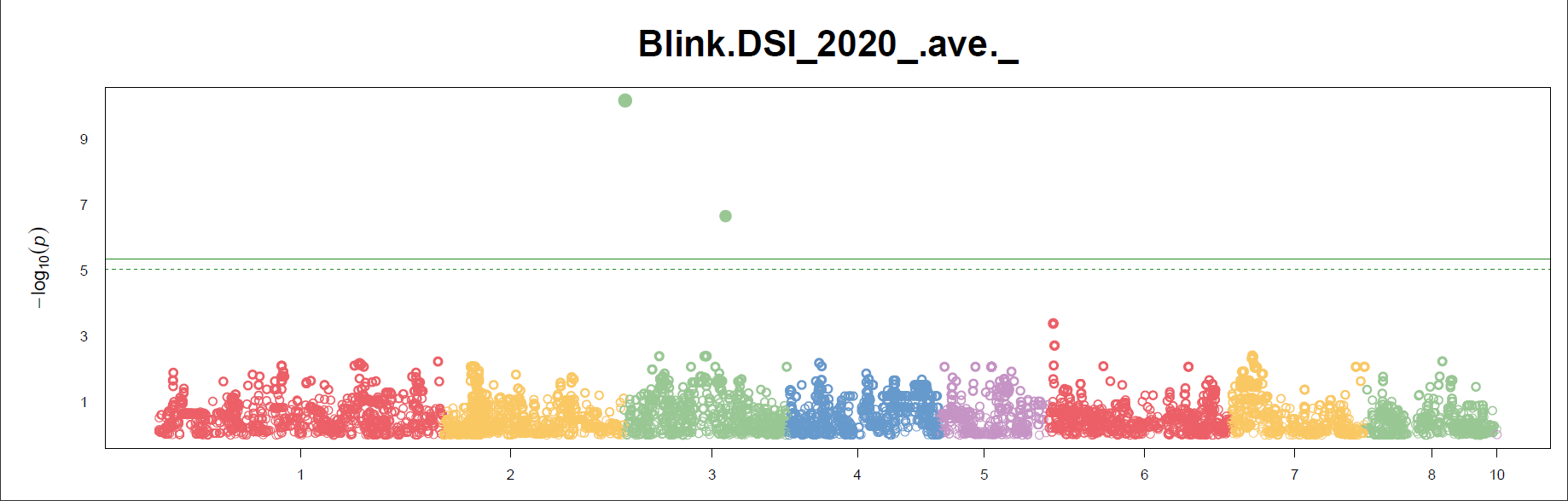 | 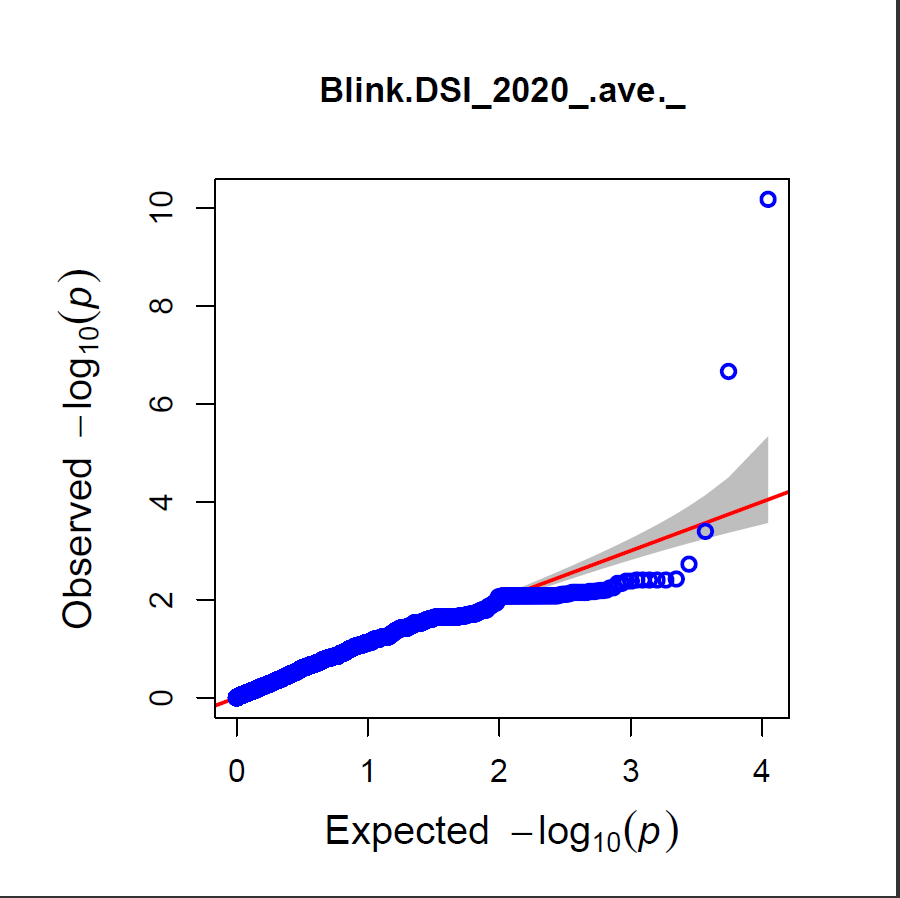 |
| 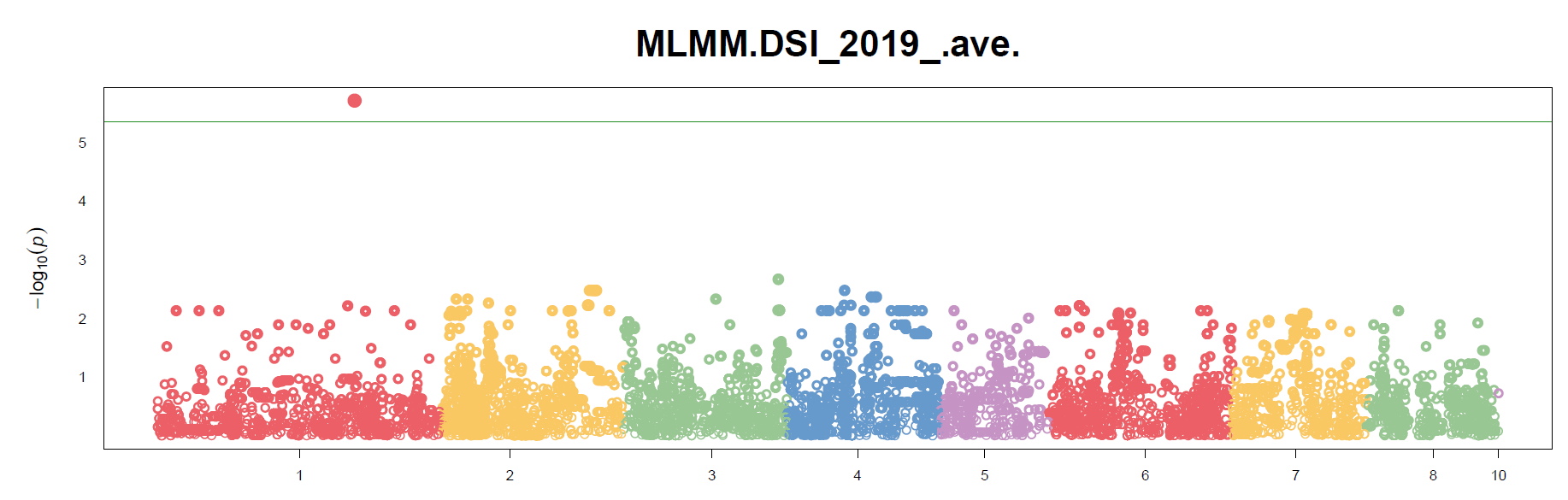 | 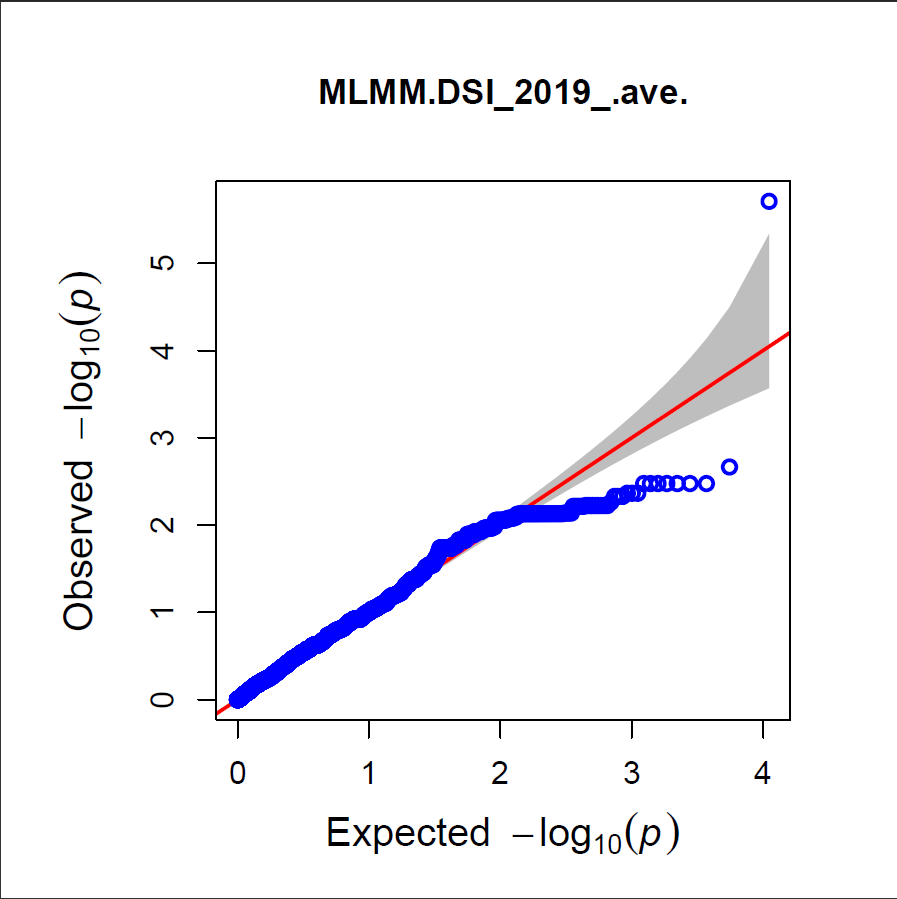 |
| 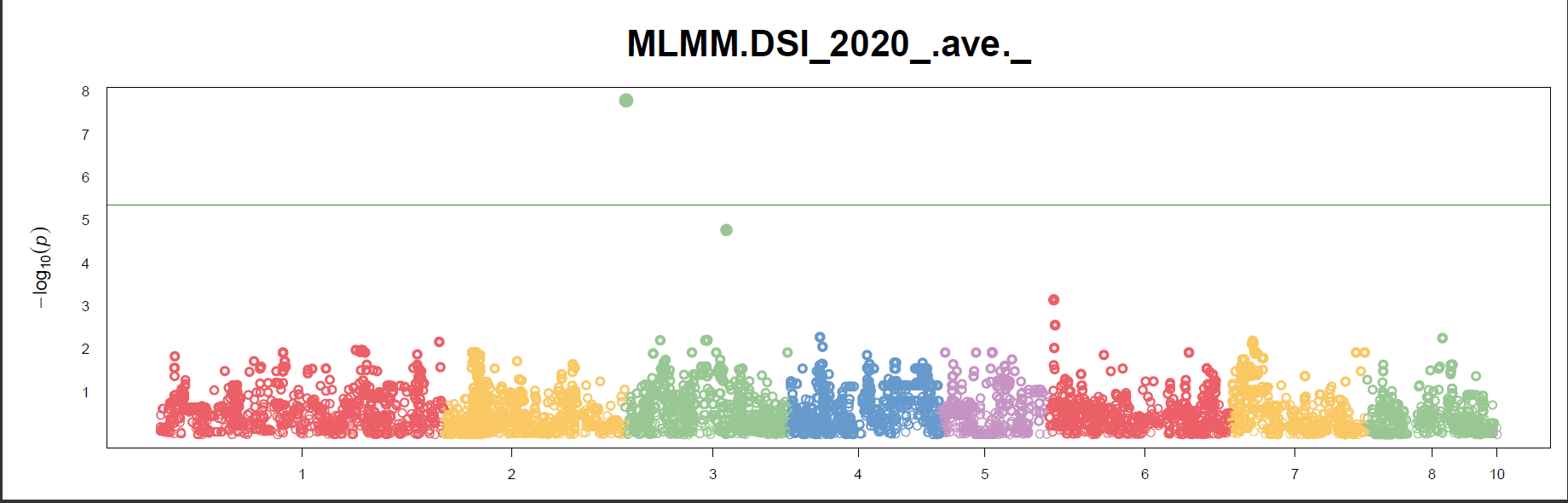 | 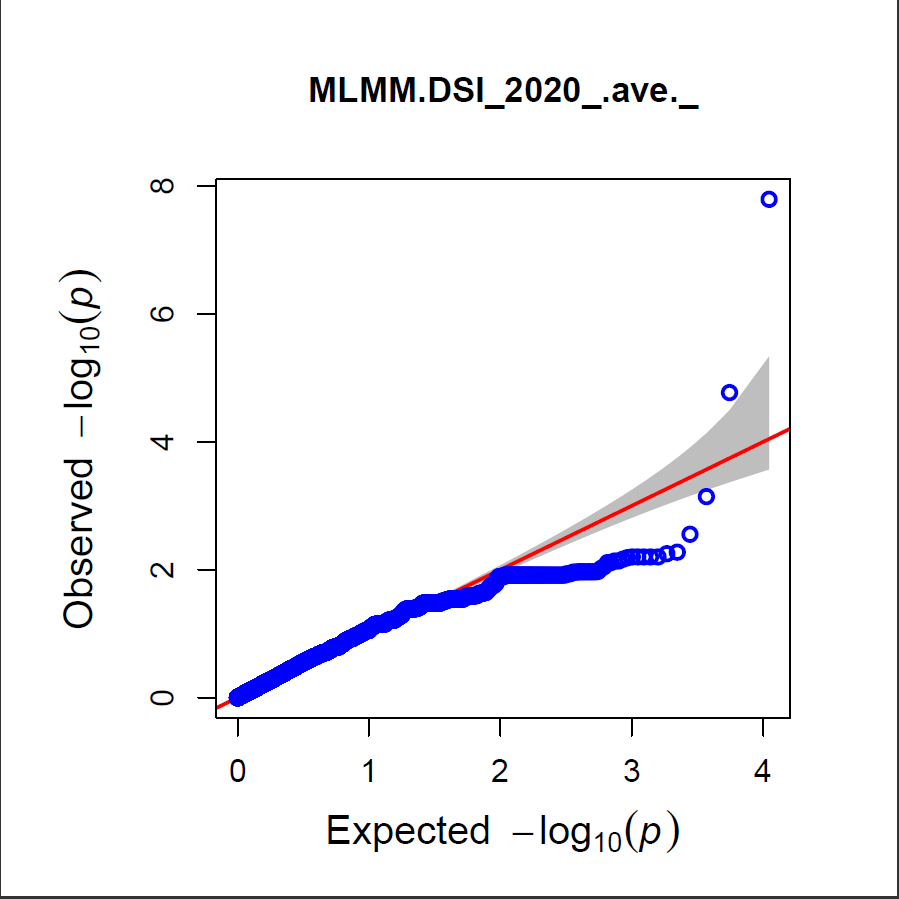 |
| 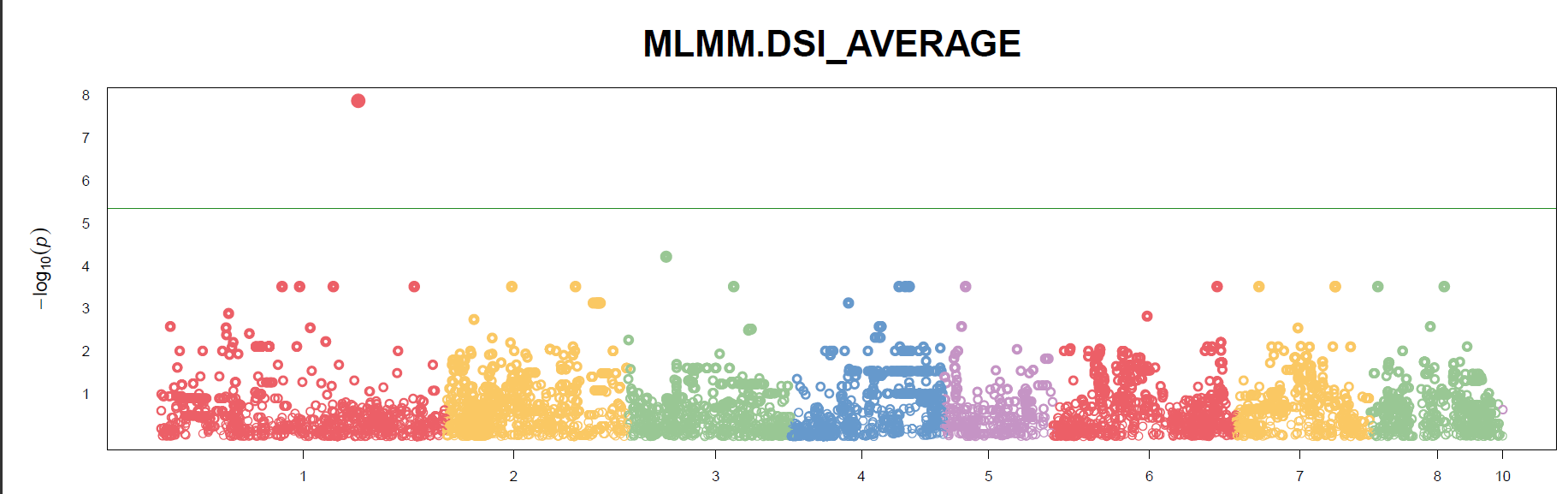 | 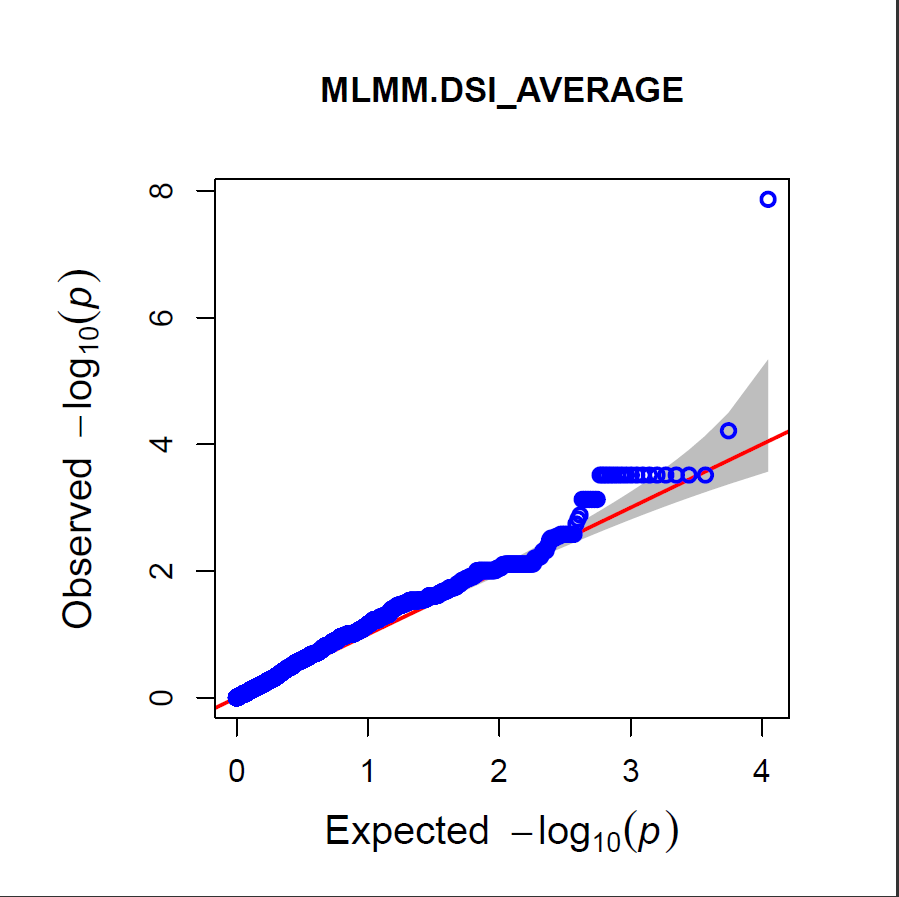 |

**Supplementary Figure 3**. Manhattan plots and QQ plots of significant SNPs obtained in GAPIT R package.

| DSI 2019 |  |
| --- | --- |
| 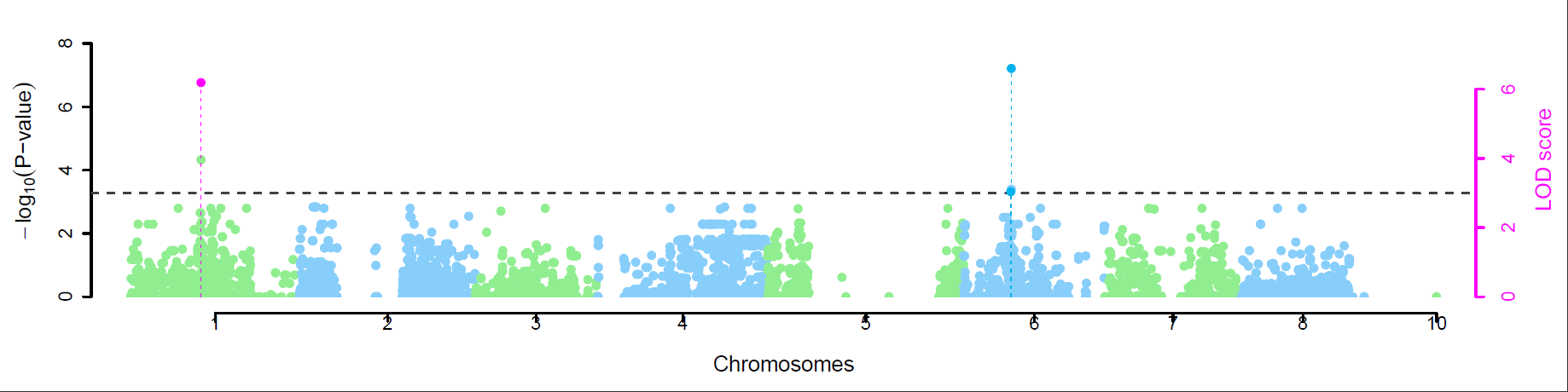 | 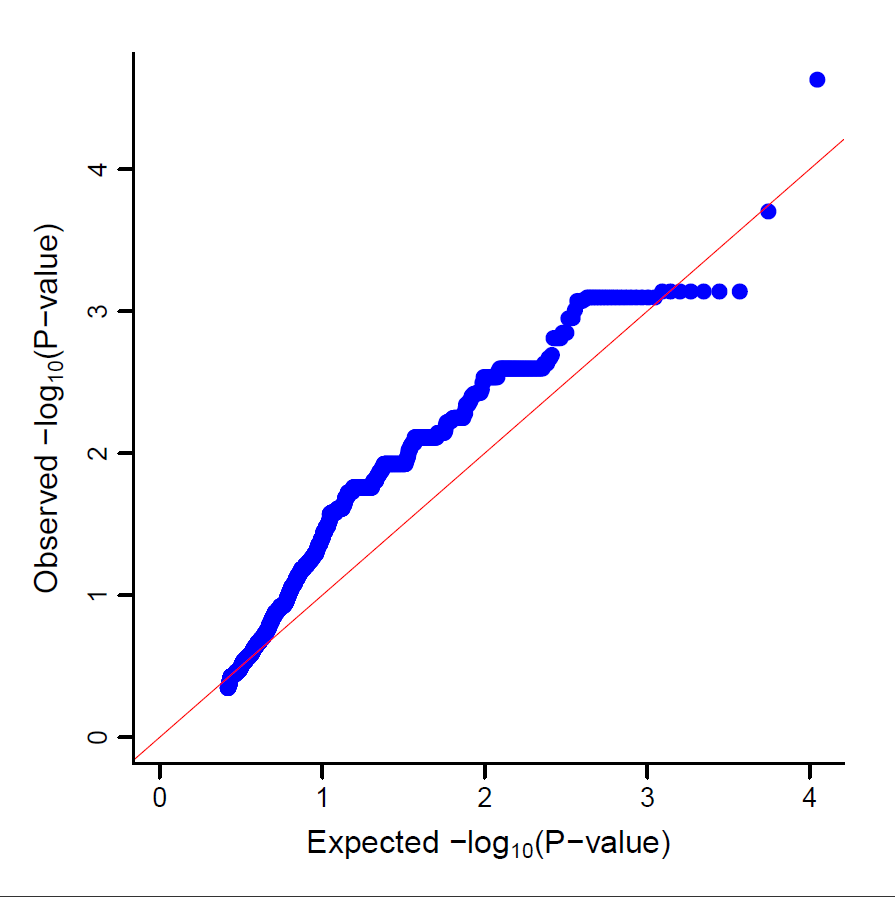 |
| DSI 2020 |  |
| 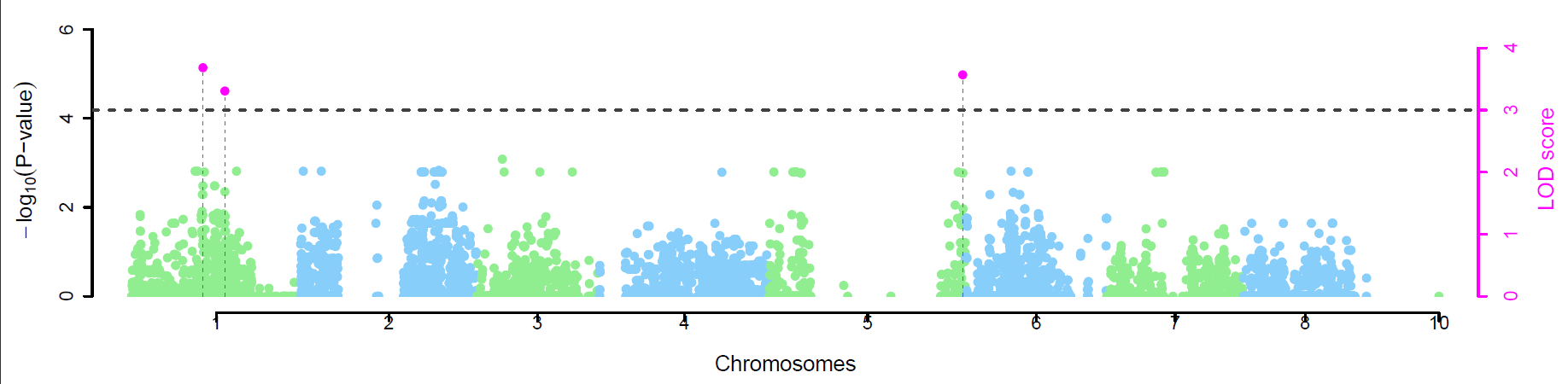 | 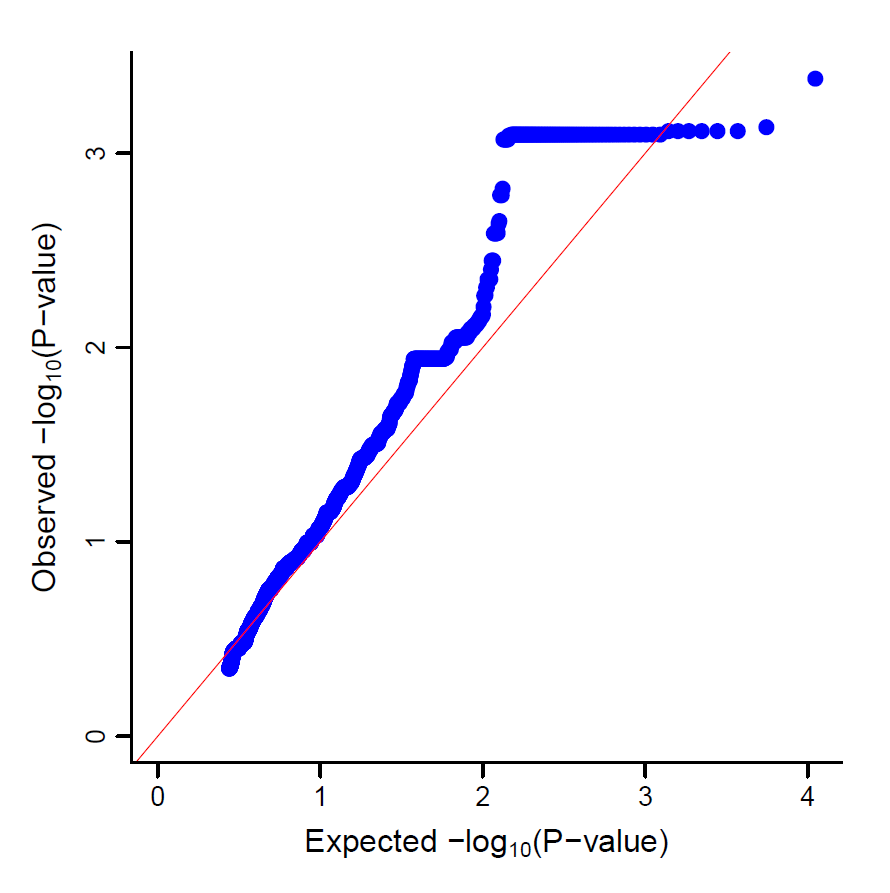 |
| DSI AVERAGE |  |
| 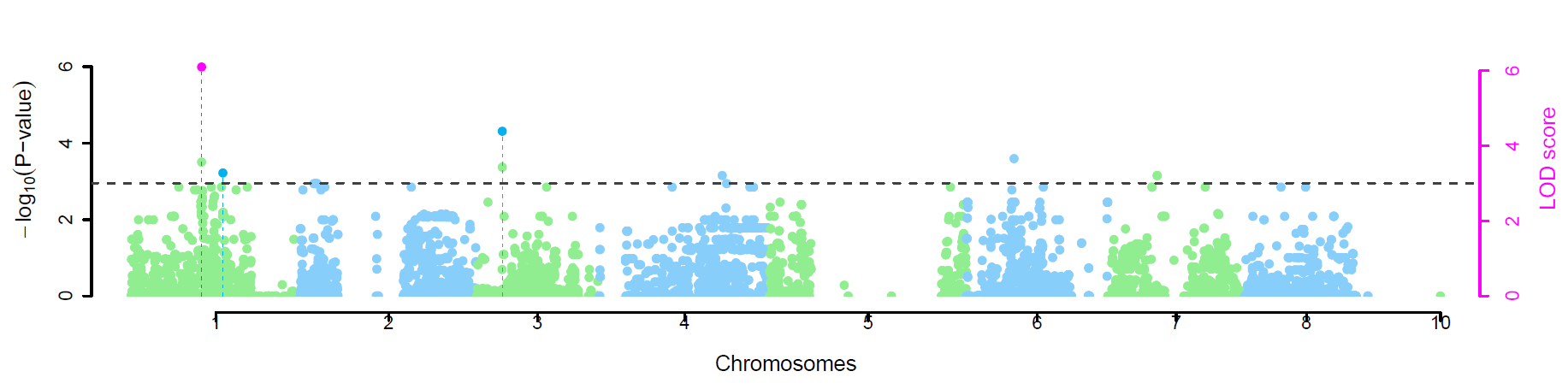 |  |
| CD 2019 |  |
| 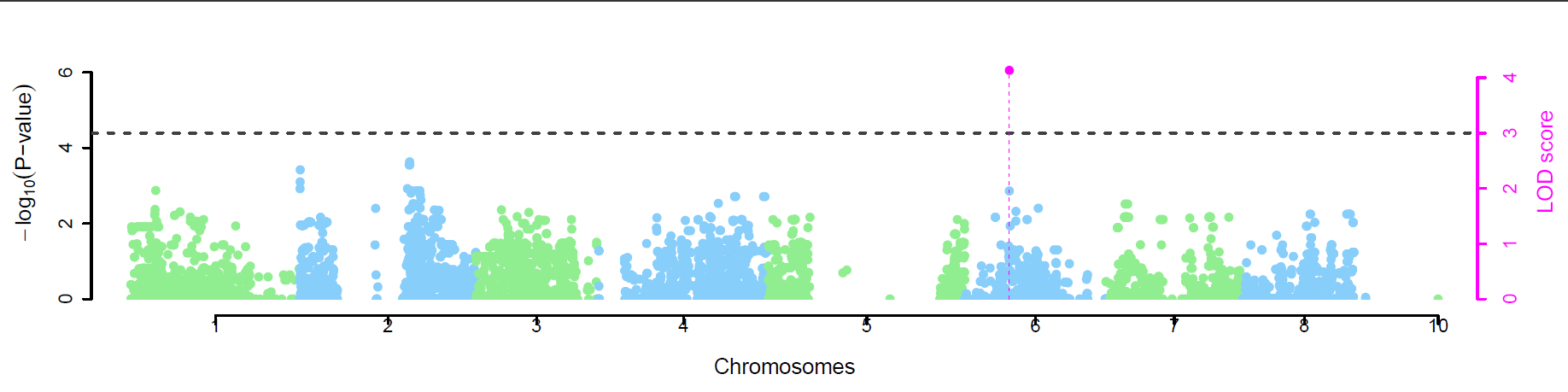 | 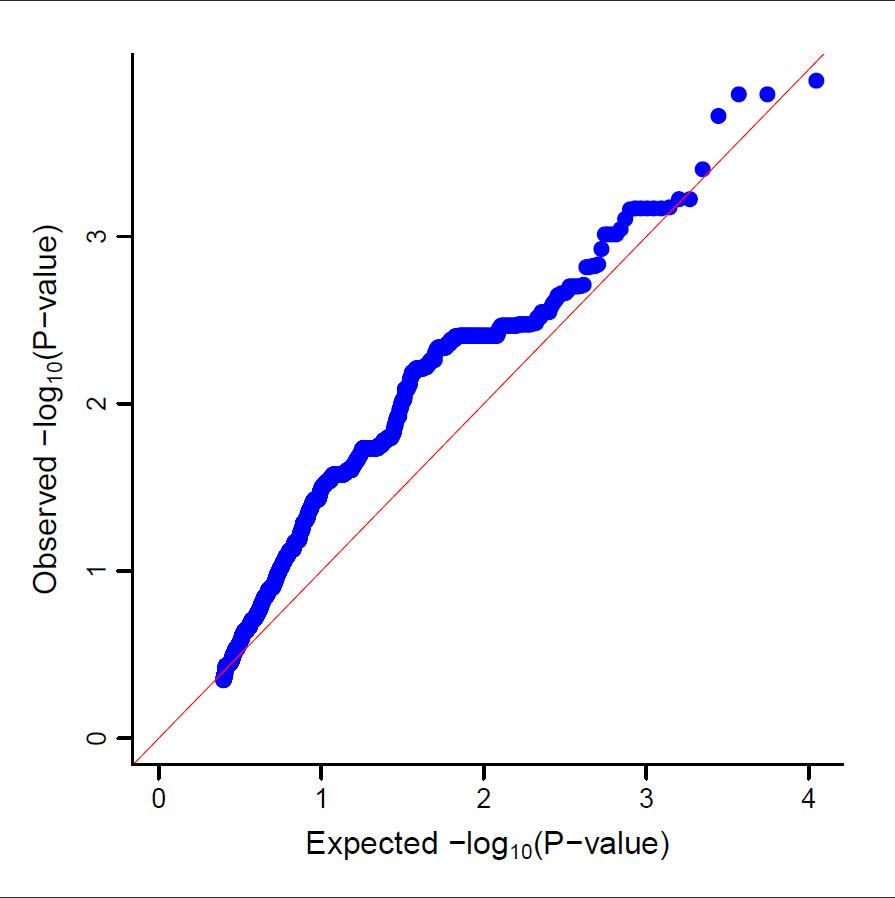 |
| CD 2020 |  |
| 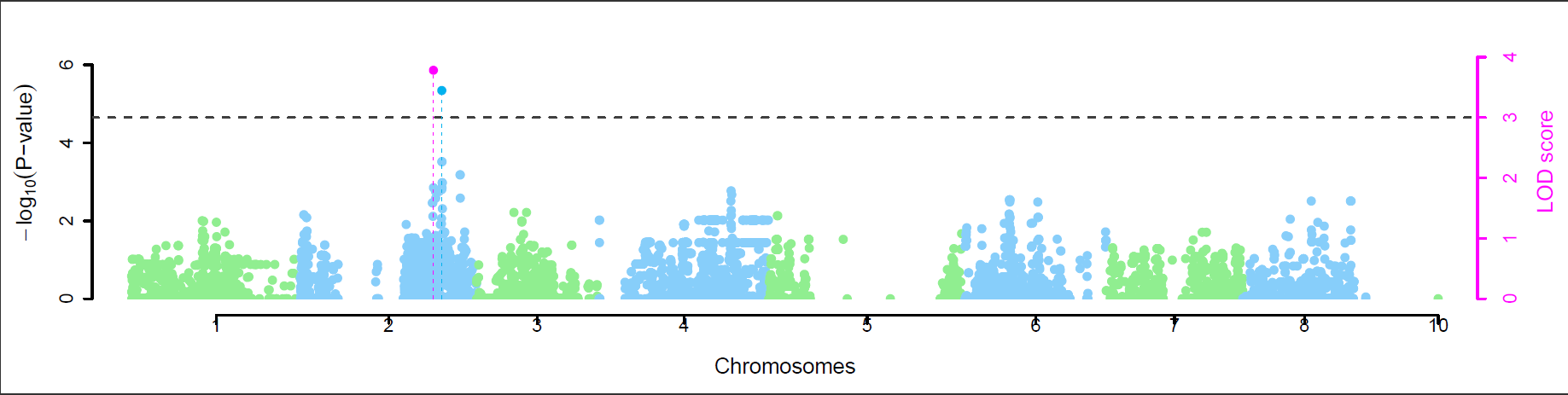 | 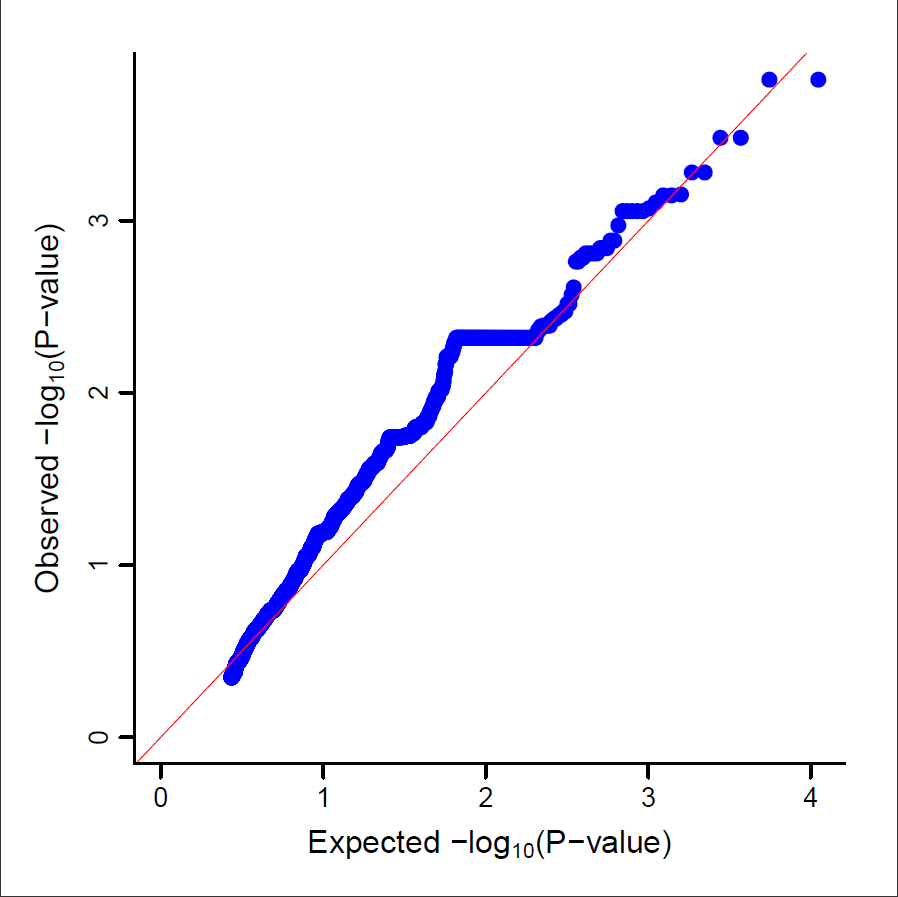 |
| CD AVERAGE |  |
| 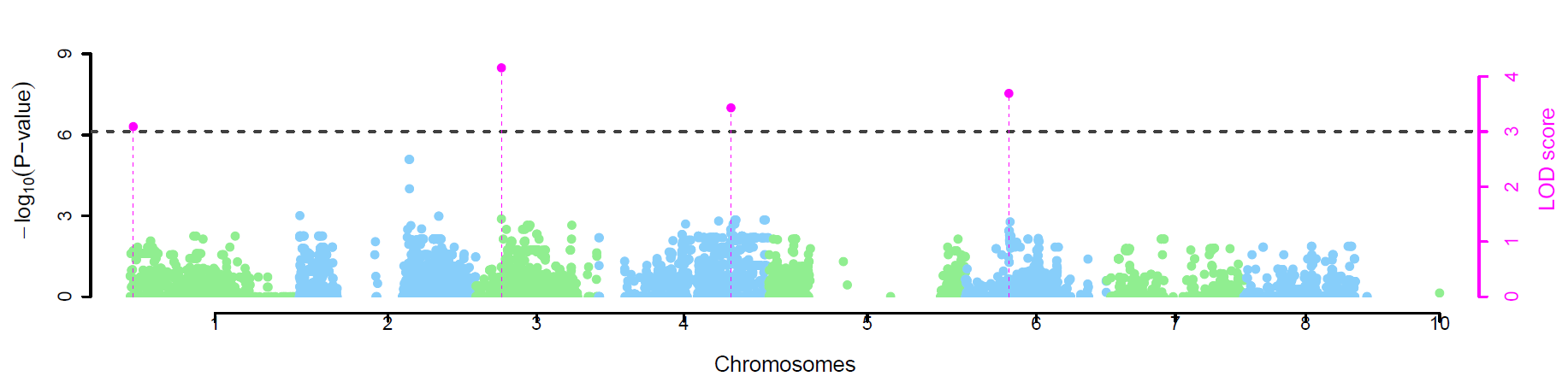 | 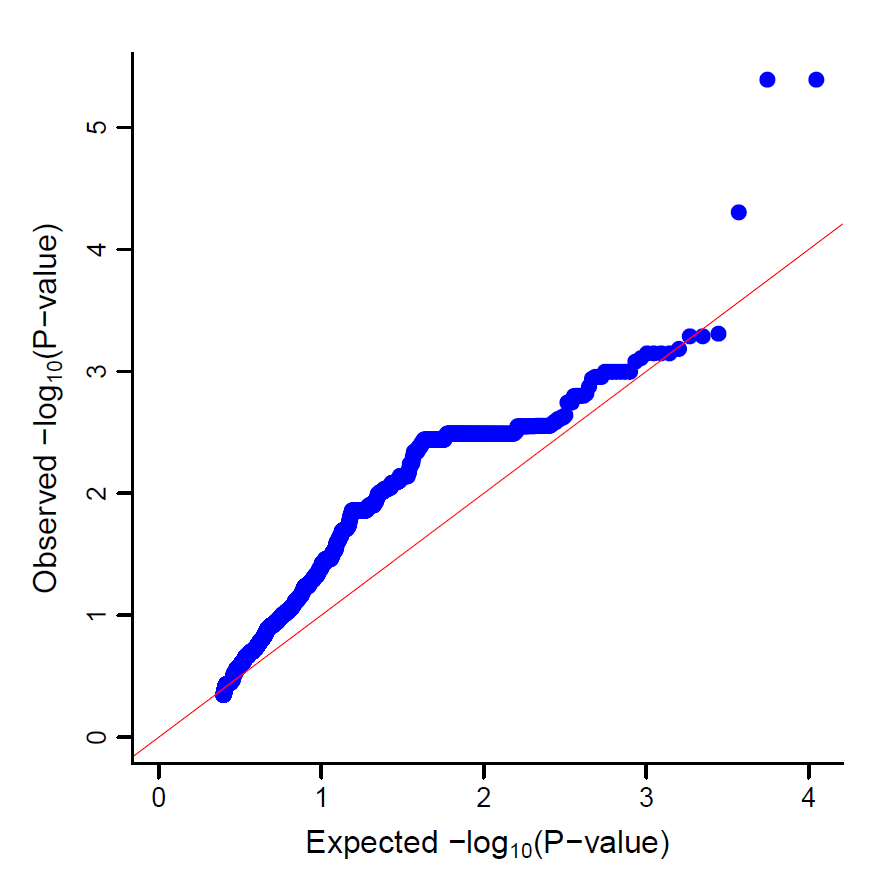 |
| CT 2020 |  |
| 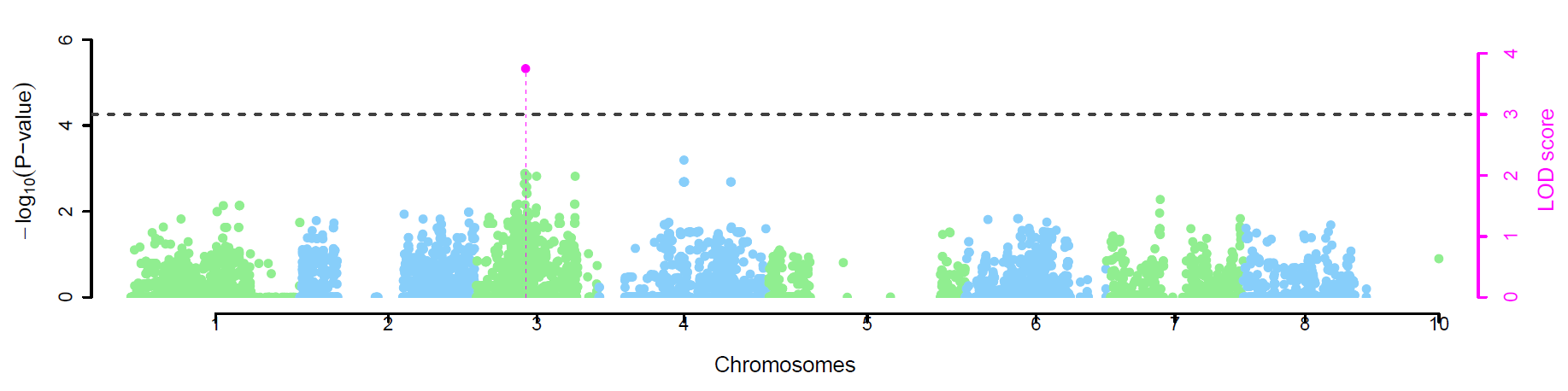 | 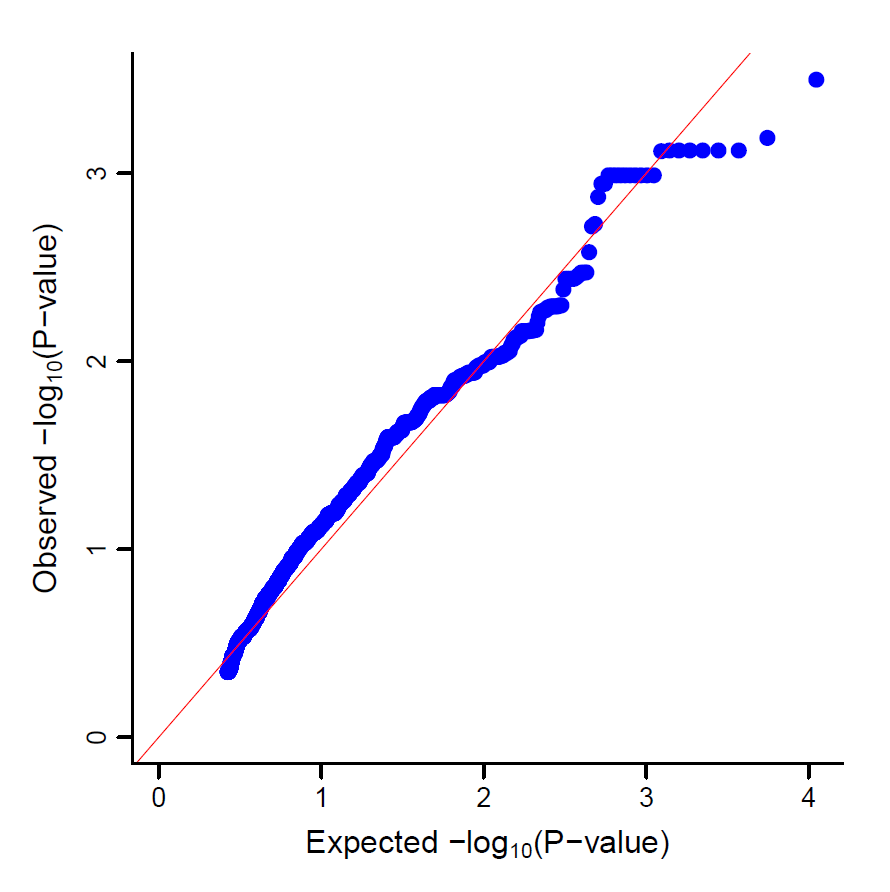 |
| CT AVERAGE |  |
| 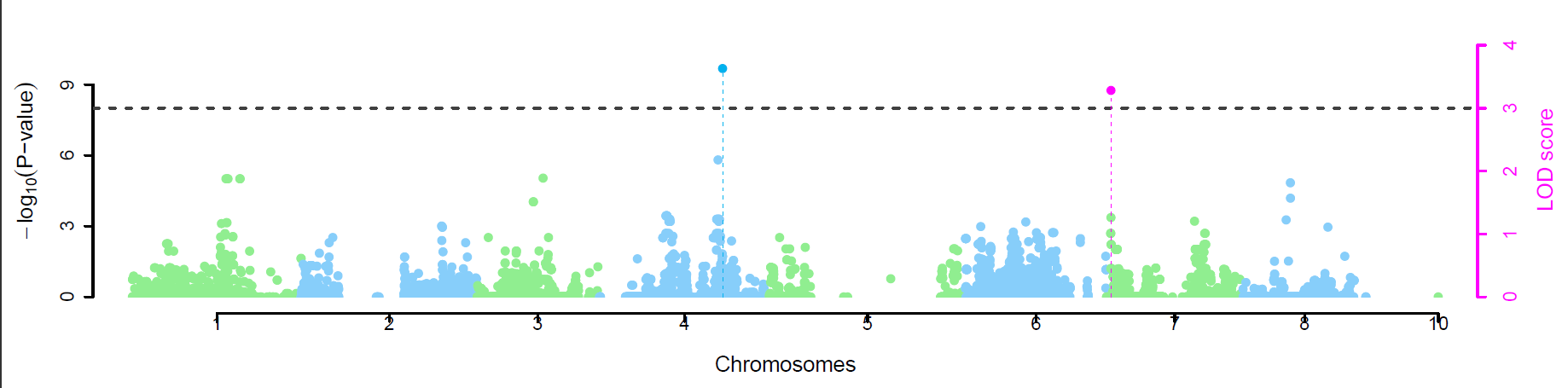 | 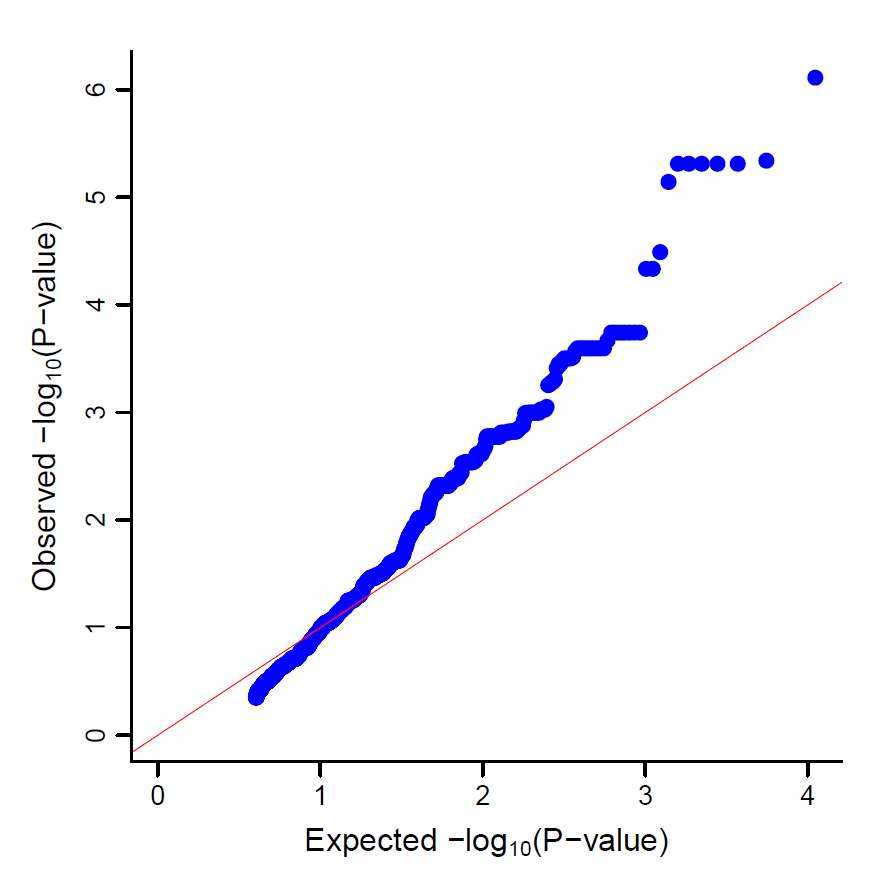 |

**Supplementary Figure 4**. Manhattan plots and QQ plots of significant SNPs obtained in mrMLM R package.

| 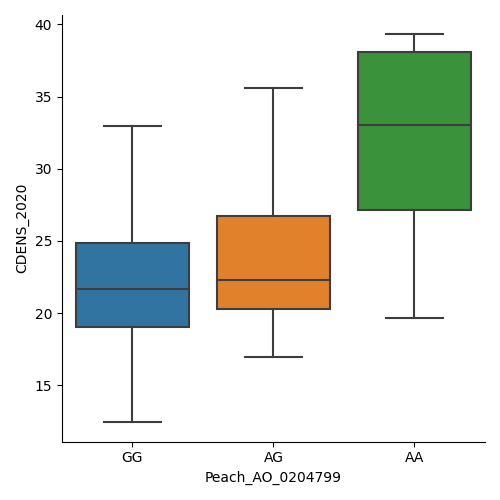 | 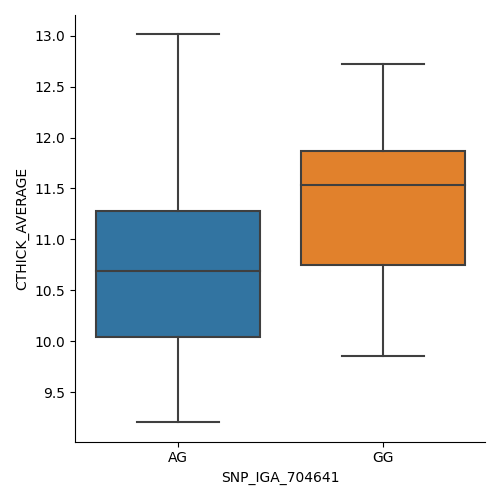 |
| --- | --- |
| 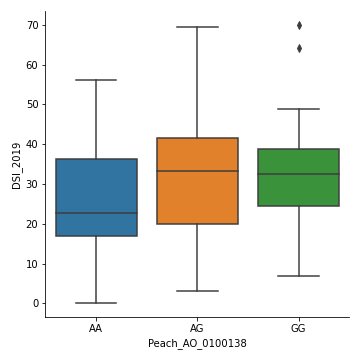 | 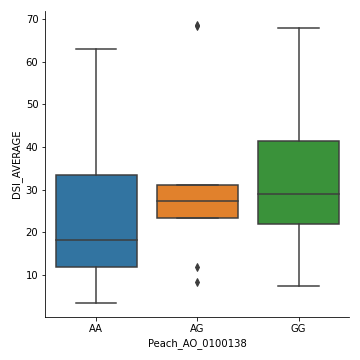 |
| 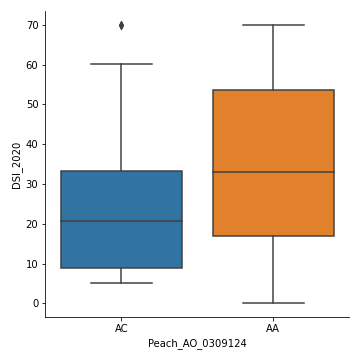 | 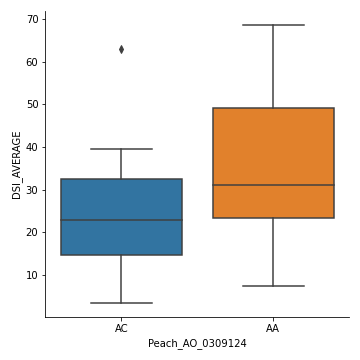 |
| 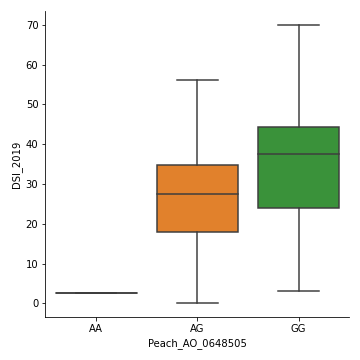 | |

**Supplementary Figure 5**. Allelic effect of the significant SNPs identified by two or more datasets/models.
